# Drug-drug interactions and QT prolongation as a commonly assessed cardiac effect - comprehensive overview of clinical trials

**DOI:** 10.1186/s40360-016-0053-1

**Published:** 2016-03-10

**Authors:** Barbara Wiśniowska, Zofia Tylutki, Gabriela Wyszogrodzka, Sebastian Polak

**Affiliations:** Unit of Pharmacoepidemiology and Pharmacoeconomics, Department of Social Pharmacy, Faculty of Pharmacy, Jagiellonian University Medical College, Medyczna 9 Street, 30-688 Krakow, Poland; Department of Pharmaceutical Technology and Biopharmaceutics, Faculty of Pharmacy, Medical College, Jagiellonian University, Medyczna 9 Street, 30-688 Kraków, Poland; Simcyp Ltd. (part of Certara), Blades Enterprise Centre, S2 4SU Sheffield, UK

**Keywords:** QT prolongation, Drug-drug interactions, Clinical trials

## Abstract

**Background:**

Proarrhythmia assessment is one of the major concerns for regulatory bodies and pharmaceutical industry. ICH guidelines recommending preclinical tests have been established in attempt to eliminate the risk of drug-induced arrhythmias. However, in the clinic, arrhythmia occurrence is determined not only by the inherent property of a drug to block ion currents and disturb electrophysiological activity of cardiac myocytes, but also by many other factors modifying individual risk of QT prolongation and subsequent proarrhythmia propensity. One of those is drug-drug interactions. Since polypharmacy is a common practice in clinical settings, it can be anticipated that there is a relatively high risk that the patient will receive at least two drugs mutually modifying their proarrhythmic potential and resulting either in triggering the occurrence or mitigating the clinical symptoms. The mechanism can be observed either directly at the pharmacodynamic level by competing for the molecular targets, or indirectly by modifying the physiological parameters, or at the pharmacokinetic level by alteration of the active concentration of the victim drug.

**Methods:**

This publication provides an overview of published clinical studies on pharmacokinetic and/or pharmacodynamic drug-drug interactions in humans and their electrophysiological consequences (QT interval modification). Databases of PubMed and Scopus were searched and combinations of the following keywords were used for Title, Abstract and Keywords fields: interaction, coadministration, combination, DDI and electrocardiographic, QTc interval, ECG. Only human studies were included. Over 4500 publications were retrieved and underwent preliminary assessment to identify papers accordant with the topic of this review. 76 papers reporting results for 96 drug combinations were found and analyzed.

**Results:**

The results show the tremendous variability of drug-drug interaction effects, which makes one aware of complexity of the problem, and suggests the need for assessment of an additional risk factors and careful ECG monitoring before administration of drugs with anticipated QT prolongation.

**Conclusions:**

DDIs can play significant roles in drugs’ cardiac safety, as evidenced by the provided examples. Assessment of the pharmacodynamic effects of the drug interactions is more challenging as compared to the pharmacokinetic due to the significant diversity in the endpoints which should be analyzed specifically for various clinical effects. Nevertheless, PD components of DDIs should be accounted for as PK changes alone do not allow to fully explain the electrophysiological effects in clinic situations.

**Electronic supplementary material:**

The online version of this article (doi:10.1186/s40360-016-0053-1) contains supplementary material, which is available to authorized users.

## Background

Cardiovascular toxicity remains one of the leading causes of early and late attrition during the drug development process as well as a major contributor to withdrawals of marketed drugs [1–3]. Cardiac safety concerns arise from a variety of side effects of the drugs including, but not limited to, direct myocyte injury, activation of apoptotic and necrotic changes, alternation of ion homeostasis or the signaling pathways or influence on the transcription factors i.e. kinase inhibitors [4, 5]. Though, proarrhythmia represents one of the most frequent cardiac safety liabilities responsible for cardiotoxic effect especially in the late stage of clinical development and during post-marketing surveillance [6]. The most important drug-induced form of proarrhythmia is acquired long QT syndrome (LQTS) and resulting potentially fatal polymorphic ventricular tachycardia termed torsades de pointes (TdP).

Tremendous progress has been made in research on and understanding of mechanisms underlying QT prolongation and TdP risk since the 1920s when quinidine syncope was first recognized. Quinidine was introduced to the practice as an antiarrhythmic for patients with atrial fibrillation [7]. Soon thereafter reports of sudden, occasionally fatal syncopal episodes occurring within therapy initiation period began to appear. It was not until the advent of online electrocardiographic monitoring that the ventricular tachyarrhythmia was described as the cause of “quinidine syncope” phenomenon in 1964 by Seltzer and Wray [8]. Characteristic for quinidine polymorphic arrhythmia was later observed by Dessertenne in a patient with atrio-ventricular block [9]. To describe his observations, twisting QRS complex around the isoelectric line on the surface ECG, he coined the term “torsades de pointes”. Interestingly, both of these early reports neither highlighted nor commented on prolonged QT interval, which was then observed in patients who were reported in the late 1970s to have developed TdP. Since that time much effort has been invested in elucidation of mechanism of drug-induced TdP and despite some reservations QT prolongation is currently recognized as an underlying cause of development of the TdP arrhythmia and thus major focus of drug development and a significant concern for regulatory agencies [10]. It is well known that QT prolongation is not directly correlated with TdP occurrence and ventricular fibrillation. Regardless of the reservations to the predictability of this marker and growing awareness of its imperfection, QT interval prolongation is still most commonly used in vivo surrogate of the proarrhythmic potency of drugs (ICH E14 guidelines). Prolongation of the repolarization process, reflected by long QT in ECG, can result from a net reduction in the outward current due to either decreased outward potassium currents (IKr or IKs), or activation of a delayed sodium current, or an increased inward calcium current [11–14]. However, most cases of prolonged repolarization related to drug exposure can be traced to the inhibition of hERG (human ether-a-go-go-related gene) potassium channel regulating major repolarizing current in the heart, IKr [15–17]. Therefore during non-clinical phase of drug development the concentration of the tested substance producing half-maximal block of the hERG potassium current (IC50) is an in vitro surrogate for proarrhythmic propensity of a compound. The class III antiarrhythmics, for which hERG inhibition underlie in part therapeutic mechanism of action, are found to carry the highest risk of TdP, estimated incidence in general population of 5 % [18]. Yet, inherent property of hERG channel is the ability to bind and interact with diverse chemical structures that encompass several therapeutic classes, including, apart from antiarrhythmics, antibiotics, prokinetics, antipsychotics and antihistamines [19, 20]. The TdP risk for non-cardiac drugs is generally estimated to be in the range of < 0.01 % up to 0.1 %, however in patients certain drugs e.g. dofetilide (anthiarrhythmic) in high doses it can be as high as 10.5 % [21]. Moreover, most of these drugs which are used for the symptomatic treatment of rather benign conditions, are more frequently prescribed with hardly any ECG monitoring [22, 23]. Upon the results of the survey conducted by De Ponti et al. it is estimated that up to 3 % of patients in the UK and Italy are prescribed at least one non-cardiac drug with proarrhythmic propensity supported by published data and official warnings about QT prolongation or TdP occurrence [24]. Estimates made by Curtis and colleagues [25] are even higher. Authors conducted retrospective study and analyzed QT-prolonging drugs prescribed with the use of outpatient prescription claims database of the largest pharmaceutical benefit in the United States and concluded that over 1 million out of about 5 million patients cohort use at least 1 QT prolonging medication (~23 % of patients). This can explain a great deal of attention and significant efforts put into the understanding, detailed screening and governing of the potential proarrhythmic potency of novel drugs by the regulatory (ICH S7 and ICH E14 guidelines), academia and industry worldwide (ICH S7 E14 guidelines).

Additionally, the problem of acquired QT prolongation and TdP is further complicated in patients undergoing polytherapy. Since polypharmacy is a common practice in clinical settings, it can be anticipated that there is a relatively high risk that a patient will receive at least two drugs mutually modifying their proarrhythmic potential and resulting in clinical symptoms or mitigating symptoms connected with one of them [22, 26–29]. In the study by Curtis and colleagues in a cohort of 1.1 million patients the concomitant use of 2 QT prolonging agents was identified in 9.4 % of patients, and the use of ≥3 agents in 0.7 % of patients. Indeed, clinical cases of TdP are frequently related to polypharmacy and drug-drug interaction resulting in QT interval prolongation [25] The results of retrospective analysis of FDA AERS (Food and Drug Administration Adverse Event Reporting System) database by Shaffer et al., where concomitant risk factors for QT prolongation and TdP occurring in association with administration of macrolide antimicrobials were examined, pointed out that co-administration of drug prolonging QT interval accounts for 50 % of registered TdP reports [30].

**Fig. 1:**
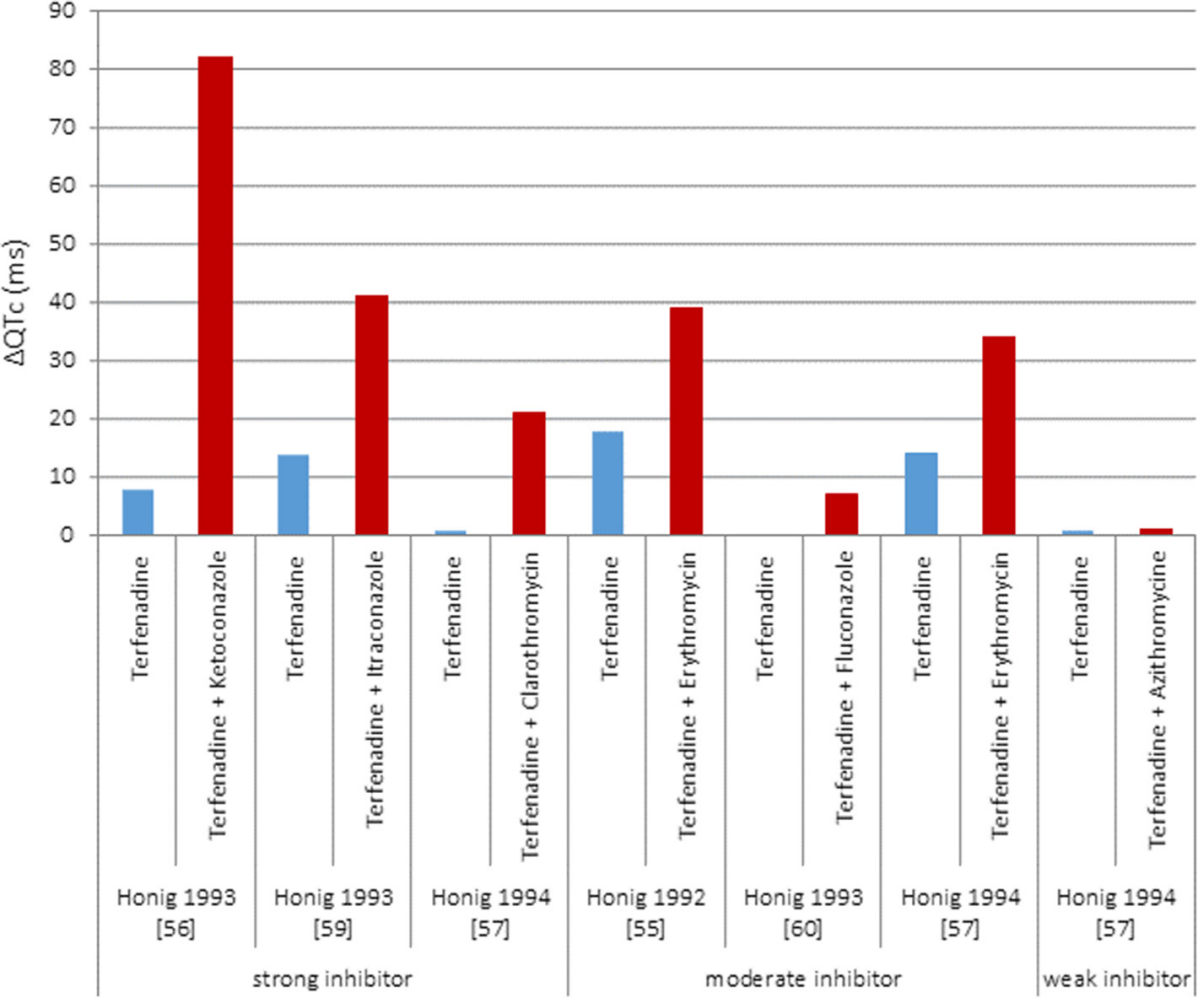
Results of five clinical studies with terfenadine given either alone or concomitantly with different CYP 3A4 inhibitors

In populations receiving multiple medications potential drug-drug interactions (DDIs) are of major concern, therefore the topic has received much attention and some formal approaches have been established. However, they concern only pharmacokinetic (PK) interactions occurring due to alterations in drug metabolism or disposition, while assessment of pharmacodynamic (PD) interactions is hardly possible due to character of the effects of such endpoints which are serious adverse effects rather than measurable changes in drug concentration [31]. Although the incidence of clinically significant PD interactions is much lower than that of PK interactions, they should not be underestimated since they can be of great importance for patient safety [32]. In case of TdP arrhythmia QT interval prolongation can be used as a relatively safe indicator of PD drug interaction result, though a number of drug combinations possibly implicated in QT prolongation is practically infinite and it is infeasible to comprehensively assess all of them during the drug development process and clinical trials. Additionally significant diversity in the study design, clinical endpoints analysis, studied populations and other factors make the PD component of DDIs challenging to analyze. Terfenadine can be used as an example due to multiple available studies clearly showing wide range of clinical endpoints, in this case QT prolongation. Figure 1 presents results of five studies where terfenadine was given either alone or concomitantly with different CYP 3A4 inhibitors. One can note obvious QT prolongation which on one hand side proves the role of DDI, yet at the same time significant variability in the effect for both scenarios (with and without inhibitor) can be also seen.

### The aim of the study

This publication provides an overview of published clinical studies results on pharmacokinetic and/or pharmacodynamic drug-drug interactions in humans (either healthy volunteers or patients) and their electrophysiological consequences manifested as change in the QT/QTc interval. The main aim was to present the role and potential clinical relevance of drug combinations for cardiac safety as evidenced in clinical trials. We aimed in presenting of the diversity of parameters influencing the electrophysiological effects of drug combinations in clinic situations; the specific statistical analysis was neither planned nor conducted.

## Methods

Databases of PubMed and Scopus were searched through November 2014 without time limit. Combinations of the following keywords were used for Title, Abstract and Keywords fields: interaction, coadministration, combination, DDI and electrocardiographic, QTc interval, ECG. Only human studies were included. Over 4500 publications were retrieved and underwent preliminary assessment to identify papers accordant with the topic of this review. Inclusion criteria met only articles describing clinical studies where the effects of two or more agents given concomitantly on the QT interval length were analyzed. All case study reports were excluded from the analysis. Reference lists from the eligible papers were the additional source of publications included in this review. Studies on combinations of antiarrhythmic drugs where their effect on QT interval was not of primary concern, and pharmacokinetics of involved compounds was out of their scope, were considered as not eligible for this review. Finally, we found 76 publications to be eligible for this review. They were divided into several groups, primarily according to the compound defined as victim drug in a clinical trial.

The results of the presented literature review are divided into two parts: 1) general description of drug classes studied for the effects of their combinations with metabolic inhibitors or other agents which might be given concomitantly; and 2) tabularized description of studies protocols, participants, PK and/or PD changes. The latter is provided as the Additional file 1: Table S1.

## Results

### Antiarrhythmics

Antiarrhythmics were the first drugs associated with QT interval prolongation and ventricular arrhythmia with the quinidine being most frequently implicated with the estimated TdP incidence in patients treated ranging from 1 to 8.8 % or even 28 % in certain groups [18]. Treatment of disorders of cardiac rhythm often requires polytherapy with antiarrhythmic drugs, many of which undergo cytochrome CYP450 metabolism. They may be inhibitors or inducers of CYP enzymes. For this reason PK and PD interactions between antiarrhythmic compounds can be of clinical importance in terms of efficacy and toxicity, especially for compounds with narrow therapeutic range, e.g. quinidine, digoxin, flecainide. Several studies involving antiarrhythmic drugs had been identified. Their results indicate that multi-drug regimens may improve treatment efficacy and safety if chosen properly. On the other hand, they point out the fact that knowledge of existing and plausible drug interactions is essential to optimize therapy for individual patients.

Twelve published reports from clinical trials were identified for antiarrhythmic drugs. Tartini and Kappenberger described excessive prolongation of QT interval and development of torsades de pointes arrhythmia in two patients treated with a combination of quinidine and amiodarone for minor arrhythmia [33]. The interaction was confirmed in a 33-year-old male healthy volunteer. Amiodarone caused significant elevation of quinidine concentration (90 % increase of C_max_) which led to substantial (41 % as compared to baseline) prolongation of QT interval [34]. The need for careful ECG and quinidine concentrations monitoring was concluded. On the contrary, subsequent administration of verapamil may have a protective effect against quinidine-related TdP arrhythmia. In the study described by Theisen and Scheininger [35] verapamil i.v. was shown to slightly (10 ms) but significantly shorten prolonged QTc interval supervene as a result of quinidine i.v. administration without lowering quinidine efficacy in a slowing flutter rate and conversion of atrial fibrillation. In most studies considering quinidine interactions it was used rather as a perpetrator than victim drug and possible victim drug toxicity or lack of its effectiveness were of primary concern instead of quinidine cardiotoxicity. In the study aimed at determining whether calcium antagonists interact with digoxin and influence its glycoside effects, quinidine (250 mg t.i.d.) induced substantial increase (118 %) in digoxin concentration [36]. The above resulted in intensification of digoxin glycoside effects and attenuation of its QTc shortening effect. Other compounds tested, namely verapamil, gallopamil, propafenone and nifedipine, amplified digoxin concentration to a lesser extent (16-77 %) and further shortened QTc interval. Two further studies investigated a role of quinidine, as a potent inhibitor of the genetically-determined debrisoquine 4-hydroxylation (CYP 2D6), in the interactions with propafenone and propranolole [37, 38].

Both studies showed significant elevations of victim drugs concentrations: propafenone mean steady-state plasma concentration increased by 169 % in a group of extensive metabolizers (no change in 2 poor metabolizing subjects) and propranolol AUC (area under the plasma drug concentration-time curve) by 92 %, moreover, the effect was stereoselective. In Funck-Brentano study despite changes in propafenone plasma concentration induced by low dose of quinidine (50 mg) electrophysiological parameters remained unaltered. The same dose of quinidine administered with propranolole resulted in QTc interval prolongation (up to 29 ms) which may be of therapeutic importance. However, explanation for this was unclear since neither propranolole nor quinidine produced delay of repolarization process when given alone. Changes in quinidine or its metabolites (not studied) and effects of propranolole and its metabolites on ventricular repolarization are among suggested explanations.

Increased arrhythmia risk is also reported to be related to high concentrations of flecainide, metabolized by the cytochrome P450 2D6 izoenzyme antiarrhythmic agent with relatively narrow therapeutic range. This gave an incentive for a study of flecainide interaction with amiodarone, a weak inhibitor of cytochrome CYP 2D6 among healthy volunteers with polymorphism of CYP 2D6 [39–41]. The study in healthy Caucasian population by Funck-Brentano et al. failed to find significant differences between flecainide concentrations in both CYP 2D6 phenotypes (poor and extensive metabolizers). Significant ECG changes were observed at flecainide dosage of 100 mg b.i.d. but not at dosage of 50 mg b.i.d., the extent of changes was similar in both phenotypes. Combination with amiodarone resulted in significant increase in flecainide plasma concentration and more pronounced QTcF (Fridericia's correction) change in both, extensive and poor metabolizers. Similarly to administration of flecainide alone, the extent of QTcF interval prolongation did not differ significantly between the groups. Dosage adjustment for flecainide was suggested if amiodarone is to be co-administered. These results differ from those reported for population of diseased infants, where both effectiveness and safety of combined flecainide and amiodarone in refractory tachyarrhythmias were demonstrated [42]. Fenrich and colleagues declare concordance of their findings with other studies conducted in adult patients with supraventricular and ventricular tachycardia. Similarly to Funck-Brentano study, Lim et al. did not find any significant differences in flecainide C_max_ at both occasions, namely administration of flecainide alone or in combination with paroxetine, between study groups of healthy Korean male subjects genetically determined as extensive (EM), intermediate (IM), and poor metabolizers (PM) [41]. QTc intervals at baseline were comparable in all 3 groups, significant increases from time-matched baseline in the QTc intervals were found in all genotype groups following flecainide administration. The extent of additional QTc prolongation, when paroxetine was added, was similar (about 6 ms) and statistically significant in EM and IM subjects. QTc prolongation in PM was less pronounced (3.9 ms). Quantitatively QTc changes in study groups did not correspond with AUC changes, and large interindividual pharmacodynamic variability is suggested as an explanation of this situation. Authors conclude that plasma flecainide concentration determination may be an insufficient predictor of electrophysiologic changes [40].

Another antiarrhythmic drug which can be used in combinations and tested for its electrophysiological consequences was tedisamil. Tedisamil, as a bradycardic agent can be used in patients with angina pectoris either alone or in combination with other anti-anginal drugs thus Demolis et al. designed and conducted a study aiming in evaluation of its effects on heart rate and QT interval duration either alone or in combination with atenolol [43]. Tedisamil, but not atenolol, prolonged QT intervals in study subjects. Observed prolongation was reverse rate-dependent as QT prolongation was much less pronounced at high rather than at low heart rates. This correlation remained unchanged during combined therapy with atenolol. Moreover, since the extent of QT prolongation during concomitant administration of atenolol and tedisamil was similar to those observed in monotherapy the authors state that there is no additional risk arising from the combination of atenolol and tedisamil.

Quinidine, a class IA antiarrhythmic agent was also tested in combination used in the therapy of condition not related with heart rhythm disorders. In the therapy of pseudobulbar affect (PBA) dextromethorphan is co-administered with low quinidine doses. While quinidine doses are of orders of magnitude lower than those for arrhythmia treatment, 20-30 mg vs 800-2400 mg [44], the related risk of QT interval should be negligible, however, during clinical trials investigating efficacy of dextromethorphan-quinidine combination in PBA [45–48] patients were monitored for electrophysiological abnormalities. The results proved the assumption on safety of low-dose quinidine therapy.

### Antihistamines

Terfenadine and astemizole were the first second-generation, non-sedating antihistamines. When launched to the market they were considered as a great breakthrough in the allergy pharmacotherapy. However, enthusiasm for them was hampered soon after by the case reports on torsades de pointes [49–52]. A growing number of alarming reports on terfenadine-related QT prolongations and TdP cases prompted the FDA to ask its manufacturer to withdraw the drug due to concerns about potential of the cardiac arrhythmias and patients sudden deaths, especially because a new, safer alternative - fexofenadine was approved. About a year later Johnson & Johnson voluntarily withdrew astemizole from the global market. The data from both astemizole and terfenadine adverse event reports submitted via the FDA AERS allowed to conclude that ventricular arrhythmia was associated with substantially elevated plasma concentrations of these drugs, resulting from the drug-drug interactions preventing its metabolic degradation, hepatic impairment, or overdose [53, 54]. This urged clinical studies that were about to test the hypothesis on the role of metabolic inhibition and pharmacokinetic changes in a QT interval prolongation following astemizole and terfenadine administration.

Honig and colleagues published a series of papers upon their research investigating terfenadine interaction with well-known inhibitors of hepatic oxidative metabolism, namely erythromycin, clarithromycin and ketoconazole, which may be co-administered [55–57]. They also conducted a study for a new at that time antifungal agents: itraconazole and fluconazole, which both demonstrate weaker in vitro inhibition of CYP enzymes than ketoconazole [34, 58], in order to investigate the magnitude of probably less pronounced but still plausible interaction [59, 60]. The aim of all the studies was to examine the influence of DDIs on terfenadine PK and its pharmacodynamic consequences represented by electrocardiographic changes. Six to nine healthy subjects, both female and male, were enrolled for each particular study. During terfenadine monotherapy (first study phase), only some individuals had detectable terfenadine concentrations (limit of quantification in plasma, LoQ – 5 ng/mL), while inhibitor was added, levels of unmetabolized terfenadine increased above LoQ depending on its CYP 3A4 blocking potency. Accordingly, fluconazole (moderate inhibitor) and azithromycin (weak inhibitor) did not cause any significant PK changes nor accumulation of the unmetabolized terfenadine. The results for azithromycin were confirmed by Harris and colleagues in 1995 [61]. In this study none of the subjects had detectable (>10 ng/mL) terfenadine plasma level throughout the whole study period. Two further studies investigated terfenadine DDI with fluoxetine and paroxetine [62, 63]. In all the studies after administration of terfenadine the QTc interval was prolonged on average by 1 to 18 ms as compared to baseline. As a consequence of changed exposure, mean QTc increased from 1 to 82 ms (azithromycin and ketoconazole) across the studies.

Another study investigated terfenadine interaction with an antidepressant nefazodone [64], which has been shown to selectively inhibit cytochrome CYP 3A4 enzymes in clinical doses [65]. Terfenadine C_max_ and AUC were markedly increased with concomitant administration of nefazodone. As expected, interaction resulted in the significant QTc intervals prolongation when compared to baseline. Two further studies investigated the PD component of interaction with terfenadine [66, 67]. They both involved sparfloxacin, a broad-spectrum fluoroquinolone antibiotic pre-clinically and clinically shown to cause dose-dependent QT interval prolongation. In a group of healthy men volunteers pharmacokinetics of sparfloxacin remained unchanged after addition of terfenadine during study by Morganroth et al. [66]. Akhtar and colleagues did not investigate PK either for terfenadine or for sparfloxacin [67]. There was no significant increase in the QTc duration in the terfenadine-treated subjects in both studies. A combination therapy was found to be connected with additive effect of concomitantly administered drugs on the QTc length.

A significant interindividual variability in ECG response to altered parent terfenadine concentrations was demonstrated in all of the above presented studies, suggesting a large role of physiological and genetic factors accounting for individual susceptibility to a QT prolongation and cardiac arrhythmia occurrence. Furthermore, substantial variability of inhibitor effect on terfenadine concentrations was observed in all studies. This may be due to the differences between subjects affecting bioavailability of the drugs or genetically determined diverse levels of CYP450 3A4 expression as well as different CYP inhibitory potencies of ketoconazole, itraconazole and erythromycin. It has been suggested that function of P-gp or OATP transporters altered by concomitantly administered drugs may influence the disposition of victim drugs, however, an interindividual variation in drug transporter expression further complicates predictions of the interaction effects in a population [68–71].

Also a single report on the effects of co-administration of terfenadine metabolite (fexofenadine) with azithromycin was found [72]. As opposed to results of terfenadine-azithromycin study, in this study co-administration of both drugs in a population of healthy volunteers resulted in substantial increase of fexofenadine bioavailability, however, it did not lead to any statistically significant or clinically relevant changes in ECG (QTc interval prolonged by 1.4 ms).

Astemizole interactions were investigated in two studies [73, 74]. Itraconazole, unlike dirithromycine, significantly increased astemizole systemic exposure. However, a QT interval change was not observed in any of the studies. Nevertheless, authors strongly suggest avoidance of concomitant administration of astemizole and itraconazole because of possible differences between single dose study results and chronic astemizole intake and anticipated effect of observed in the Lefebvre study astemizole clearance reduction for prolonged astemizole therapy [74]. Also Bachmann advised further investigation of electrophysiological consequences for astemizole interaction proposing study with different design, however, since Johnson & Johnson withdrew astemizole from the market two years after Lefebvre and Bachmann studies, and according to our best knowledge, no additional investigations with publicly available results were carried out [73].

The awareness that the cardiotoxicity is not a class effect of antihistamines, yet their administration can induce the QT prolongation and in certain circumstances be potentially proarrhythmic prompted further studies aimed at assessment of individualized risk for some antihistaminic compounds. One of the drugs extensively studied for its interactions and their electrocardiographic effects is loratadine with its active metabolite desloratadine.

Four papers reporting five clinical trials with loratadine administered concomitantly with CYP enzymes inhibitors having different inhibitory profiles were identified [75–78]. The influence of potent CYP 3A4 (clarithromycin, ketoconazole, nefazodone), moderate CYP 3A4 (erythromycin), and weak CYP 3A4 and 2D6 (cimetidine) inhibitors, according to the FDA classification [34], on loratadine PK had been studied. The data from those studies demonstrated anticipated effects of applied inhibitors on the clearance of both loratadine and desloratadine. Despite the observed differences between magnitude of the pharmacokinetic interactions of loratadine with diverse CYP inhibitors the pharmacodynamic consequences were principally the same. The tested drug combinations were safe and well tolerated. The mean QTc interval changes for drug combinations were not significantly different from the increase accompanying co-administration of inhibitors with placebo. The results of majority of studies suggest that loratadine does not share the proarrhythmic potential of other nonsedating antihistamines, in particular terfenadine, and considering the wide safety margin of loratadine its interactions with CYP inhibitors are probably clinically unimportant.

Safety profile of desloratadine, a major metabolic derivative of loratadine, has been confirmed in three independent clinical studies with both recommended clinical and supratherapeutic dose in healthy volunteers [72, 79–82]. It was demonstrated that despite induced by ketoconazole, erythromycin, and azithromycin, higher desloratadine exposure, the co-medications were well tolerated and caused neither significant QTc interval prolongation nor changes in adverse events profile. The authors unanimously concluded that desloratadine combinations with CYP inhibitors are safe and may be administered to the patients without concerns about clinically significant cardiac events.

Tyl and colleagues report on results of the ICH E14 guideline compliant thorough QT/QTc study for a bilastine [83]. It was shown that bilastine at therapeutic and supratherapeutic doses don’t have any impact on QTc intervals measured in healthy volunteers. Co-administration with ketoconazole resulted in an increased bilastine bioavailability and resulted in increased systemic exposure. Clinically significant increase in QTc intervals duration was observed, however, this was most likely related to ketoconazole effect alone, as bilastine concentrations did not exceed those during supratherapeutic doses administration and no correlation between bilastine PK and QTc was found.

The results of studies concerning antiallergics show that the actual QT prolongation differs substantially between individuals, and is dependent not only on hERG inhibition potential of the agent and metabolic interactions resulting from polytherapy but also other stages of ADME process can significantly influence proarrhythmia risk.

### Gastrointestinal prokinetic agents

Cisapride, a gastric pro-motility agent indicated for gastro-oesophageal reflux disease treatment, is another, after terfenadine and astemizole, hallmark example of market withdrawal of non-cardiac blockbuster drug due to unacceptable risk of a QT interval prolongation and TdP arrhythmia. Cisapride was removed from the global market [84] as a consequence of several case reports of major cardiac toxicity, following analysis of the FDA AERS database by Wysowski et al. 2001 [85] and a case-control study [86] which provided evidence that cisapride is associated with the increased occurrence of the QT prolongation, ventricular arrhythmia and sudden cardiac death. It is worth noted that apart from the drug related characteristics Wysowski noticed the role of other factors influencing cisapride clinical effect, including but not limited to certain health conditions (i.e. heart diseases, electrolytes disorder). However, in spite of its withdrawal, cisapride is still available in the U.S. through an investigational program to patients who meet certain criteria since it is considered to be the most effective agent for gastric motility, as well as in some European countries. Administration of cisapride alone is known to be a cause of the QT prolongation [87–90]. However, many of the adverse reactions ascribed to cisapride occurred in patients with conditions that could predispose to cardiac arrhythmias or taking other drugs that inhibit cisapride metabolism via CYP 3A4 enzymes or prolong QT interval [91]. The increased risk following metabolic inhibition and co-administration of other QT-prolonging drug was demonstrated by van Haarst [92] and Zix [93]. Interestingly, concomitant therapy with clarithromycin caused substantially greater change in the QTc interval duration than would be expected from simple additive effect of both drugs. Due to hERG blocking properties of both drugs [94] it was conceivable that PD interactions may occur with this combination. Nonetheless, van Haarst study design did not allow for quantitative assessment of PK and PD component contribution in this interaction, but dependence of the QTc intervals on cisapride concentration was observed what suggests an overriding role of PK component in the overall electrophysiological effect. The second study investigated the clinical relevance of possible cisapride interaction with QT-prolonging drug - sparfloxacin. Considering that, as it was proven in the study, cisapride has no influence on sparfloxacin PK and assuming that there is no influence of sparfloxacin on cisapride PK, the observed QTc prolongation can be ascribed to PD interaction only. Nevertheless, reported results do not allow to assess whether the type of the interaction is additive or synergistic.

Cisapride has narrow therapeutic index thus even weak inhibition of CYP mediated metabolism and consequent modest concentration increase is potentially important. As depression and gastroesophageal reflux disease often occur together and many of SSRI (selective serotonin reuptake inhibitors) demonstrate some extent of CYP inhibitory properties, it is important to determine whether potentially harmful interactions occur between antidepressants and cisapride. In the two identified studies fluoxetine [95] and sertraline [96] were studied for effects of their interaction with cisapride. Data from both studies indicate that cisapride can be safely administered to patients who are treated with fluoxetine and sertraline while neither fluoxetine nor cisapride, nor sertraline, nor their combination induced statistically or clinically significant ECG parameters change. This was due to decreased cisapride concentrations caused by both drugs. For sertraline the above is in accordance with the results of studies concluding that it is a weak inducer of CYP enzymes [97, 98], however for fluoxetine reasons for such an effect are unclear, while fluoxetine with its active metabolite, norfluoxetine, are inhibitors of CYP P450 system enzymes [99].

Two newer, more selective (as compared to cisapride) 5-HT4 receptor agonists were studied for their cardiac safety under conditions known to favor arrhythmia development with cisapride, namely CYP mediated metabolism inhibition [27, 100, 101]. The results of both studies indicate that neither mosapride nor cinitapride itself carry proarrhythmic potential since they did not produce any changes in the electrocardiograms. Also, there was no significant correlation between plasma concentrations of both drugs and QTc intervals. Moreover, concomitant administration with potent CYP inhibitors, ketoconazole and erythromycin, resulted in only slight increases in plasma concentrations of victim drugs, though without any clinically significant changes in electrocardiographic findings.

Boyce and colleagues designed a study to assess the PK and electrophysiological effects of domperidone and ketoconazole, an agent influencing domperidone metabolism and P-gp dependent disposition, and the consequences of their interaction in healthy volunteers [26]. Ketoconazole significantly increased domperidone plasma concentrations in all subjects, however observed PD effects were strongly dependent on gender, and were more pronounced in men, for both compounds given alone and their combination. Based on the results the authors conclude that domperidone and ketoconazole should not be administered concomitantly.

### Antiemetics

Droperidol (dopamine D2 receptor antagonist) and ondansetron (serotonine 5-HT3 receptor antagonist) are the first-line antiemetics for prevention and treatment of postoperative nausea and vomiting (PONV). Both drugs have shown effectiveness in monotherapy, however, there is evidence that patients at high risk of emesis can benefit from their combination [102, 103]. Since both droperidol and ondansetron are known to prolong QT interval, it can be anticipated that the clinical effect of combined therapy may be modified by drug interactions and result in the increased risk of proarrhythmia. Two studies considering the effects of droperidol and ondansetron (alone and in combination) on QT interval duration have been identified [104, 105]. Both studies led to conclusion that arrhythmia risk for drug combination is not higher when compared with both antiemetics administered alone.

Ondansetron electrophysiological safety was also studied in combination with sevoflurane [106].

The results of this study indicated that despite of significant QTc prolongation by sevoflurane and enhancement of this effect by ondansetron the dispersion of ventricular repolarization is not affected and drug combination may be clinically safe. However, an additional risk factors assessment before administration and careful ECG monitoring was advised.

QT prolongations precipitated by droperidol and “black-box” warning issued by FDA on possible ventricular arrhythmias associated with droperidol administration led to reduction of its use and search for compound sharing its antiemetic activity but with better safety profile. Two of the considered droperidol substitutes, found to have an effect on PONV (haloperidol and midazolam), were assessed for efficacy and safety in combination with dexamethasone. No significant QTc changes were found while the incidence of PONV was reduced [107, 108].

### Psychotropic drugs

Polypharmacy involving psychotropic drug combinations is common despite the lack of evidence of its efficacy and safety [109]. The electrophysiological effects of psychotropic drugs used in monotherapy have been studied extensively, however the influence of combined therapy on QTc was not as widely investigated.

In the retrospective study, Correl and colleagues investigated the risk of QTc prolongation for patients on mono- and polytherapy with antipsychotics [110]. Patients treated concurrently with two atypical antipsychotics were matched with controls on monotherapy for sex and antipsychotic agent. The average QTc dispersion observed in combination therapy group was similar among patients treated with monotherapy, despite the fact that patients receiving antipsychotic polytherapy were administered significantly higher chlorpromazine-equivalent dosages. These results suggest that polytherapy with two atypical antipsychotics, in moderate doses, does not involve significant QTc prolongation and challenge the common assumption about dose-dependence of QTc prolongation for antipsychotics [111, 112]. Similarly, Sala with coworkers in their retrospective study did not find any significant increase in the average QTc interval following antipsychotic monotherapy (haloperidol, olanzapine, risperidone or clozapine) in female patients. There was, however, meaningful QTc prolongation noted when antidepressants (escitalopram, citalopram, mirtazapine, paroxetine, sertraline, fluvoxamine, venlafaxine, clomipramine) or lithium were administered concomitantly [113]. The average QTc interval and QTc change after treatment was 421 ± 20 ms and -1 ms in the monotherapy group and 438 ± 30 ms and 24 ms in the polytherapy group. Moreover, there was a significant difference between the treatment groups in the number of patients who had QTc values above 450 ms, seven patients (38 %) in the polytherapy group vs. one patient (7 %) in the monotherapy. Antidepressant agents investigated in the study are known to have a mild inhibitory activity on drug-metabolizing enzymes from CYP family, however, in this study PK interactions did not seem to contribute substantially to QTc prolongation in the group with combined therapy as serum levels of antipsychotics were not higher in this group compared to monotherapy patients. In the view of the results authors suggest an accurate monitoring of the QTc before and after the treatment, especially for those receiving multiple psychoactive agents with QT prolonging propensity.

Some of the drug combinations analyzed in poly- and monotherapy were also investigated more thoroughly in other studies [96, 114–117]. Drug pairs studied included other psychoactive agents commonly used together with antipsychotics and with metabolic inhibitors, administration of which is likely to coincide with psychoactive compounds (itraconazole, ketoconazole, clarithromycin). The studied DDIs did not result in as dramatic changes of PK and PD as in case of terfenadine and ketoconazole, yet complex and individualized risk to benefit ratio evaluation is advised prior to prescription of any antipsychotic.

### Agents used in drug dependence therapy

Racemic methadone is used in maintenance therapy of opiate addicts. Both S- and R-form of methadone inhibit the cardiac potassium channel hERG (S-methadone is more potent blocker) [118], which may increase heart risk. Indeed, the incidences of torsades de pointes arrhythmia in methadone-treated patients have been reported [119]. Drugs tested for potential increase in the cardiac risk of methadone include voriconazole, which can be administered concomitantly in case of serious fungal infections occurring in addicts [120]. Administration of lofexidine, an agent used inter alia for opioid detoxification, may coincide with methadone as it supports transition from methadone to buprenorphine [119]. No significant changes in QTc length were observed neither in patients receiving only methadone nor in patients on drug combination.

Another agent used in opioid dependence treatment is buprenorphine, which can be also given concomitantly with naloxone [121, 122]. Although buprenorphine is said to inhibit IKr potassium current only in concentrations higher than therapeutic, being CYP 3A4 substrate it is prone to interact and consequently to put patients at risk of QT interval prolongation. Electrophysiological consequences of CYP 3A4 inhibition induced by antiretrovirals with range of inhibitory potential were evaluated and proved this mechanism to be out of clinical importance in terms of QTc interval prolongation.

Modafinil is considered as a potential treatment for cocaine addiction, thus the co-administration of both compounds to cocaine addicts seemed possible. While cocaine is said to prolong QT interval and the potential enhancement of that effect by modafinil is probable, a study assessing ECG changes in case of concomitant administration of these agents was designed [123]. In general, this group of drugs is not associated with significant QT prolongation risk, even in combinations with agents which administration likely coincides.

### Antimalarials

To date malaria still claims the lives of people in tropical countries. Chloroquine that used to be antimalarial of choice is no longer as highly effective as initially [124]. However, it may be still used to potentiate the curative effect of other drugs. The drugs combinations investigated for their pro-arrhythmic potential include i.e. primaquine plus chloroquine, tafenoquine and chloroquine, amodiaquine and halofantrine, halofantrine and mefloquine, atovaquone and proguanil, artesunate and mefloquine, artemether and lumefantrine, dihydroartemisinin and dihydroartemisinin/mefloquine combination, dihydroartemisinin and piperaquine what gives a flavor of its potential clinical meaning with regard to the cardiac safety [125–137]. Despite substantial changes in drugs PK observed in some of the studies in a majority of cases there was no correlation between plasma concentration of drugs and electrophysiological effects and no clinically important QTc interval prolongation cases were found. Cardiac safety of antimalarials was also confirmed in a study by Lefèvre and colleagues [138] where despite the artemether/lumefantrine pharmacokinetic changes caused by ketoconazole, ECG parameters, including QTc interval, did not exceeded normal limits in either of treatment groups.

### Varia

Several interaction studies focusing on the cardiac safety of drugs from other therapeutic groups were identified. They concern anesthetics given concomitantly with anticholinergics [139], neuromuscular blockers and their antidotes [140, 141], medications used for treatment of asthma and chronic obstructive pulmonary disease (fluticasone furoate/vilanterol) [142], combination of antihypertensive drugs (losartan and spironolactone) [143] and others [29, 142, 144].

## Discussion and conclusions

Fatal in consequences terfenadine – ketoconazole case with cardiac related deaths played a significant role in recognition of the clinical role of drug-drug interactions and their desirable and undesirable consequences [145]. The pharmacokinetic DDI studies are now part of the drug screening procedure for multiple reasons which include, but are not limited to, assessment of necessity of a dosage adjustment, need for additional therapeutic monitoring, or due to the safety reasons when certain drugs should be contraindicated for concomitant use for the sake of patient safety.

**Fig. 2:**
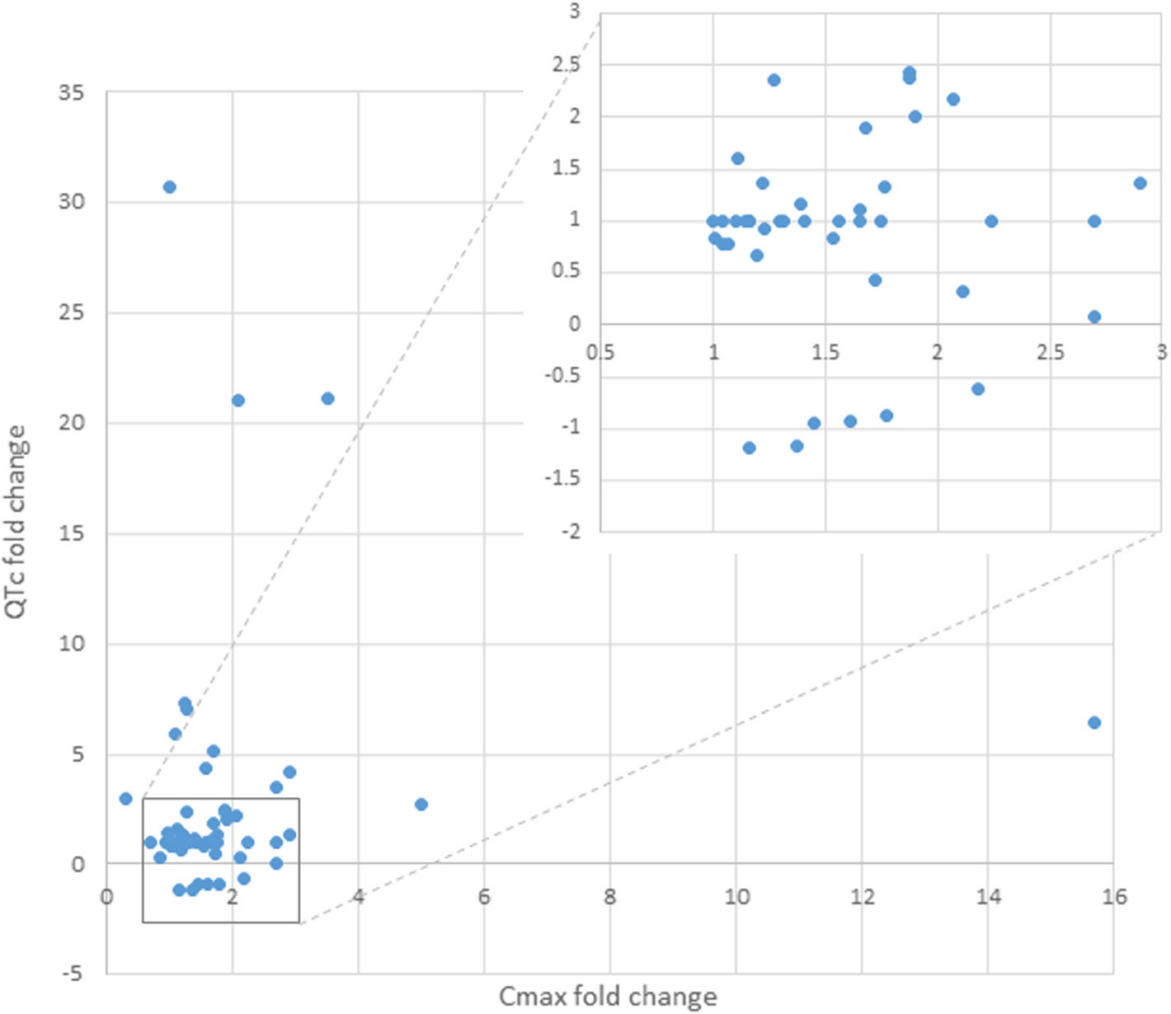
PK fold change (Cmax) vs. PD fold change (QTc). Fold change = C_max_Combination/C_max_Drug and ΔQTcCombination/ΔQTcDrug; Negative values of QTc fold change represent QTc interval change for negative ΔQTc values as compared to baseline (QTc shortening for single drug, as well as for the combination)

The available guidance focuses on the PK drug interaction studies where the blood or tissue concentrations are modified by the disruption of drug’s absorption, distribution, metabolism, and excretion processes triggered by the interacting compounds [146]. It was not assessed in the current study as it needs separate, specifically designed analysis but it is not surprising that majority of the above mentioned drugs are metabolized by CYP 3A4 and CYP 2D6 [147]. It opens the possibility to undergo the metabolic interaction (inhibition or induction) resulting in the clinical effect modification. Therefore this can be considered as a drug related risk factor, similarly to the drug transporters (e.g. P-gp) affinity, protein binding, high lipophilicity and consequent significant heart tissue penetration [148–150]. Assessment of the pharmacodynamic effects of the drug interactions is much more challenging due to the significant diversity in the endpoints which should be analyzed depending on the clinical effect. Pharmacokinetic parameters modification including AUC and C_max_ are relatively easy to assess as compared with the pharmacodynamic endpoints for which the clinical surrogates are often imperfect if they exist at all. Another challenge comes with the results variability, which results from exposure variability and factors specific for the observed parameter, so the interacting drugs add additional level of complexity to already complex situation. Analyzing trials’ results for evaluation of DDIs role in proarrhthmia risk brings additional hurdles. This is mainly due to the differences in study design e.g. single dose or multiple dose protocol, study duration, population age, sex, or ethnicity and methods of evaluation of raw data used to generate reported endpoints. First, there are several methods for QT interval length correction for heart rate, Bazzet, Fridericia or individual or population-based, just to mention those most commonly used, without individual data it is impossible to compare the results between the studies [151]. Second, the reported endpoint can be substantially influenced by the applied method of comparison. Drug-triggered QTc changes may be referred to placebo control or to the baseline QT values. Baseline measurement can be defined as QTc interval length at single point registration (rarely reported time of a day) or mean from several time points. Similarly, reported QTc values were derived in various way, e.g. maximal and/or average of individual QTc values at defined time point after drug application, maximal and/or average of individual QTc values over several hours following last day of drug application [152]. Finally, in some cases, reader can be confused about the true value of the reported endpoints, while there are different values reported in the tables and in the text or graph. Nevertheless, DDIs’ role was clearly shown in the retrospective analyses done by multiple authors. Haugaa and colleagues correlated QT-related mortality with multiple factors and found that number of QT-prolonging medications was a significant predictor of death [153]. Similar results were observed in DeBruin study where the risk of cardiac arrest was more pronounced in patients receiving more than 1 QT-prolonging drug simultaneously [154]. The collation of studies identified for this overview suggest the need for assessment of an additional risk factors for proarrhythmia as there are examples where pharmacokinetic changes do not contribute substantially to QTc interval prolongation and plasma concentrations are not correlated with observed electrophysiological effects (Fig. 2). Careful ECG monitoring before administration of drug combinations with anticipated QT prolongation, especially with concomitant risk factors, has been suggested in many of the reports. However, there are some concerns on monitoring cost effectiveness [155].

Moreover, it can be safely assumed that the list of studies presented in the current publication covers just small number of potential combinations and additionally the incidence of the life threatening situations is low therefore it is quite likely that clinical trials with their current methodology cannot offer the expected power. Also, in case of elderly, multimorbid, or critically ill patients undergoing polytherapy the DDI risk is substantially increased, while such scenario testing is barely feasible, if not impossible at all and can be only analyzed retrospectively [156]. One of the possible ways of thorough data analysis is a traditional “top-down” PK/PD modeling and simulation (M&S). In such approach empirical or descriptive models are utilized to describe the linkage between drug concentration and observed clinical response including cardiovascular biomarkers [157]. There are multiple examples of studies utilizing such approach for DDI assessment [158], although there are just few where PK/PD type of analysis was utilized for the assessment of QT modification triggered by the combination of drugs [159]. There are examples of successful implementation of nonlinear mixed-effects PK/PD models and Bayesian methods. Such approach can be especially useful when there is either limited or noised dataset. Prior distributions for the model parameters derived from previous single drug studies can improve predictivity and model quality [160].

It all suggests the need for a system of early prediction of the potential DDI clinical consequences. To the authors’ best knowledge there is no established in vitro methodology offering such possibilities therefore potential solution lays in the proper use of the in silico based methods. This proposition can be supported by the recent wide incorporation of the in silico realized in vitro – in vivo extrapolation approach to the assessment of the clinical role of drug – drug interactions [161, 162]. Recently discussed drug cardiac safety assessment paradigm change includes wide use of mathematical models of human heart cells. This fact allows us to suggest that after proper validation such methods could be also applied for the fast and cost-effective DDI consequences assessment which cannot be done in the traditional way due to the multiple obstacles.

## Additional file

Additional file 1: Table S1.
**Characteristics of clinical trials assessing electrophysiological consequences of drug-drug interactions.** (DOC 292 kb)

## Abbreviations

AUC: Area under the plasma drug concentration-time curve
C_max_: Maximal plasma concentration
DDI: Drug-drug interaction
ECG: Electrocardiogram
AERS: Adverse Event Reporting System
ICH: International Conference on Harmonization
LQTS: Long QT syndrome
PD: Pharmacodynamics
PK: Pharmacokinetics
QTc: QT interval corrected for the heart rate
TdP: Torsades de pointes
