# Supplementary material for: Drug-drug interactions and QT prolongation as a commonly assessed cardiac effect - comprehensive overview of clinical trials
Source: BMC Pharmacol Toxicol. 2016 Mar 10;17:12. doi: 10.1186/s40360-016-0053-1 (PMC4785617; doi:10.1186/s40360-016-0053-1)
Supplement: Additional file 1: Table S1. — Characteristics of clinical trials assessing electrophysiological consequences of drug-drug interactions. (DOC 292 kb) [file 40360_2016_53_MOESM1_ESM.doc]

| **AUTHOR** | **STUDY DESIGN** | **DRUG 1** | **DRUG 2** | **STUDY POPULATION** | | | | | | **PK CHANGE %** | | | **CORRECTION METHOD**  **ΔQT_c_ [ms]** |
| --- | --- | --- | --- | --- | --- | --- | --- | --- | --- | --- | --- | --- | --- |
|  |  |  |  | **No of subjects** | **Mean age [Y] ±SD and/or (range)** | **M/F** | **race** | **Weight [kg]**  **±SD and/or (range)** | **Height [cm]**  **±SD and/or (range)** | **C_max_** | **t_max_** | **AUC** |  |
| **ANTIARRHYTHMICS** | | | | | | | | | | | | | |
| Tartini 1982^33^ |  | (Q) Quinidine  3x 400 mg  (for 6 days) | (A) Amiodarone  3x 200 mg | 1 | 33 | 1/0 |  |  |  | Q: 90 |  |  | **none**  41% combination  Q: 20% |
| Theisen 1983^35^ |  | (Q) Quinidine  320 mg i.v. | (V) Verapamil  5 mg i.v. | 8 patients | 43.4 |  |  |  |  | PK changes not studied |  |  | **Bazett**  68 ms combination  Q: 78 ms |
| Belz 1983^36^ | randomized, placebo controlled,  cross-over | (D) Digoxin  3x 0.125 mg | (V) Verapamil  3x 80 mg/120 mg | 36 | (20-33) | 36/0 |  | (56-105) |  | D: 77/61 |  |  | **Bazett**  -34/-30 ms combination  D: -30/-28 ms |
|  |  |  | (N) Nifediine  3x 10 mg |  |  |  |  |  |  | D: 45.3 |  |  | **Bazett**  -32 ms combination  D: -30 ms |
|  |  |  | (G) Gallopamil  3x 50 mg |  |  |  |  |  |  | D: 15.6 |  |  | **Bazett**  -33 ms combination  D: -28 ms |
|  |  |  | (P) Propafenone  3x 150 mg |  |  |  |  |  |  | D: 36.6 |  |  | **Bazett**  -36 ms combination  D: -31 ms |
|  |  |  | (Q) Quinidine  3x 250 mg |  |  |  |  |  |  | D: 118.3 |  |  | **Bazett**  -19 ms combination  D: -31 ms |
| Zhou 1990^37^ | randomized,  cross-over | (P) Propranolol  80 mg (single dose) | (Q) Quinidine  50 mg (single dose) | 6 | 28.7±8.2 | 6/0 |  | 62.1±9.7 |  |  |  | P: 91.5 | **Bazett**  29 ms after 3 h combination  D: Not significant  P: Not significant |
| Funck-Brentano 1989^38^ | EM – extensive metabolizers of debrisoquine  PM – poor metabolizers of debrisoquine | (P) Propafenone  3x 150 mg  (for 4 days)  (5OH-P) – propafenone metabolite – 5-hydroxypropafenone | (Q) Quinidine  3x 50 mg  (for 4 days) | 9  diseased | 49.3 (40-70) | 4/5 |  |  |  | P EM: 170 (mean interdose plasma conc)  5OH-P EM: – 48.3  P and 5OH-P PM: no change |  |  | **NA**  Not significant combination |
| Funk-Brentano 1994^39^ | randomized,  open-label,  cross-over | (F) Flecainide  2x 50 mg or 2x 100 mg  (for 5 days) | (A) Amiodarone  2x 200 mg  (for 5 days) | 12 | 25.8 (23-37) |  |  |  |  | F: EM 11.1  F: PM 24.8 |  |  | **Fridericia**  EM  10.1 ms combination (with 100 mg F)  F: 1.7 ms (100 mg)  F: 6.8 ms (200 mg)  PM  8.8 ms combination (with 100 mg F)  F: 1.2 ms (100 mg)  F: 5.5 ms (200 mg) |
| Lim 2010 (PD)^40^  Lim 2008 (PK)^41^ | open-label,  cross-over  EM- CYP2D6 extensive metabolizers  IM – CYP2D6 intermediate metabolizers  PM – CYP2D6 poor metabolizers | (F) Flecainide  200 mg  (single dose) | (P) Paroxetine  1x 20 mg  (for 7 days) | EM 7  IM 7  PM 7 | EM 24.3±3.3  IM 25.3±3.3  PM 23.9±2.5 | 21/0 | A 21 | EM 173±4.5  IM 172.4±4.3  PM 175.3±5.0 | EM 66.3±7.3  IM 67.7±8.2  PM 73.3±6.1 | F EM: 22  F IM: 11.4  F PM: 16.1 | F EM: -33.3  F IM: 0  F PM: -25 | F EM: 27.9  F IM: 16.5  F PM: -1.6 | **Fridericia**  EM 23.9 ms combination  F: EM 17.4 ms  IM 17.8 ms combination  F: IM 11.1 ms  PM ~20.5 ms combination  F: PM 20.5 ms  **Individual**  EM 22.3 ms combination  F: EM 15.4 ms  IM 14.9 ms combination  F: IM 9.1 ms  PM 20.3 ms combination  F: PM 16.4 ms |
| Demolis 1997^43^ | randomized, placebo-controlled,  double-blind,  cross-over | (T) Tedisamil  2x 100 mg  (for 5 days) | (A) Atenolol  2x 50 mg  (for 5 days) | 10 | 26±3.6 | 10/0 |  |  |  | T: -7.1  A: 15.5 | T: 0  A: 0 | T: -0.5  A: 20.1 | No correction  **At rest**  45 ms combination  T: 44 ms  A: Not significant  QT=A-B*exp(-C*RR) correction for QT measured during exercise  **During exercise**  -8 ms combination (RR=500 ms)  T: 19 ms (RR=400 ms) |
| Panitch 2006^45^ | randomized, double-blind,  placebo controlled | (D) Dextrometorphan  2x 30 mg  (for 85 days) | (Q) Quinidine  2x 30 mg  (for 85 days) | 76 | 46.3±9.8 | 15/62 | B 5  A 1  H 2  W 68 |  |  | PK changes not studied |  |  | **NA**  7.5 ms combination |
| Pioro 2010^46^ | randomized, double-blind, placebo-controlled,  parallel-group | (D) Dextrometorphan  1x 30 mg  (for 12 weeks) | (Q) Quinidine  1x 10 mg  (for 12 weeks) | 110  patients | 53.1±11 | 46/64 | B 6  H 21  W 80  O 3 |  |  | PK changes not studied |  |  | **Bazett**  3 ms combination 30 mg D  **Fridericia**  4.8 ms combination 30 mg D |
|  |  | (D) Dextrometorphan  1x 20 mg  (for 12 weeks) | (Q) Quinidine  1x 10 mg  (for 12 weeks) | 107  patients | 50.8±11.1 | 53/54 | B 2  H 21  W 80  O 4 |  |  | PK changes not studied |  |  | **Bazett**  -1.9 ms combination 30/20 mg D  **Fridericia**  1 ms combination 30/20 mg D |
| Brooks 2004^47^ | randomized, double-blind, placebo controlled, cross-over | (D) Dextrometorphan  2x 30 mg  (for 28 days) | (Q) Quinidine  2x 30 mg  (for 28 days) | 65 | 54.8 ±12.8 | 42/23 | B 2  W 58  H 5 |  |  | D: 1753.9 mean conc  Q: 87.5 |  |  | **NA**  7.5 ms combination |
|  |  | Dextrometorphan  2x 30 mg  (for 28 days) |  | 30 | 53.8±11.2 | 16/14 | W 25  H 3  O 2 |  |  |  |  |  |  |
|  |  |  | Quinidine  2x 30 mg  (for 28 days) | 34 | 55.3±9.5 | 18/12 | W 31  H 3 |  |  |  |  |  |  |
| Schoedel 2012^48^ | open-label, randomized, parallel-group | (D/Q) Dextrometorphan+ Quinidine  2x 30 mg/30 mg  (for 8 days) | (P) Paroxetine  1x 20 mg  (for 12 days) | 27 | 33.6 (19-55) | 21/6 | B 12  W 10  H 4  A 1 | 74.3±10.4 | 172  (calculated from BMI) | P: 13.5  D: 41.4  Q: 29.9 | P: 0  D: 0  Q: 0 | P: 33.6  D: 46.1  Q: 38.5 | **NA**  Not significant in all groups |
| ANTIHISTAMINES | | | | | | | | | | | | | |
| Bergstrom 1997^63^ | prospective cohort study,  multiple dose | Terfenadine  1x 60 mg (single dose on day 1 and 14)  (F) Terfenadine metabolite-Fexofenadine | (Fl) Fluoxetine 1x 60 mg  (for 9 days from day ) | 12 | 42 (25-55) | 12/0 |  | 71 (56-95) | 173 (163-185) | T: -30  F: 0.1 | T: 9.4  F:15.8 | T: -42.4  F: 14.3 | **Bazett**  5.7 ms combination  T: 5.7 ms |
| Honig 1992^55^ | prospective cohort study,  multiple dose | Terfenadine  2x 60 mg (for 7 days)  (F) Terfenadine metabolite-Fexofenadine | (E) Erythromycin  3x 500 mg  (for 7 days) | 9 | 26.78 (19-42) | 4/5 |  | 73.67 (55-89) |  | F: 107 | F :81.8 | F: 170 | **Bazett**  39 ms combination  T: 18 ms |
| Honig 1993^56^ | prospective cohort study,  multiple dose | (T) Terfenadine  2x 60 mg (for 7 days)  (F) Terfenadine metabolite-Fexofenadine | (K) Ketoconazole  2x 200 mg  (for up to 7 days) | 6 | 27.67 (24-35) | 4/2 |  | 72.17 (67-89) | 171.5 (160-180) | F: not significant |  | F: 57.2 | **Bazett**  82 ms combination  T: 8 ms |
| Honig 1994^57^ | prospective cohort study,  multiple dose | (T) Terfenadine  2x 60 mg (for 7 days)  (F) Terfenadine metabolite-Fexofenadine | (E) Erythromycin  3x 500 mg | 6 | 33.8 (24-40) | 4/2 |  | 82.5 (67-103) | 177.3 (168-185) | F: 87.4 | F: 83.3 | F: 109.4 | **Bazett**  34 ms combination  T: 14 ms  E: 21.5 ms |
|  |  | (T) Terfenadine  2x 60 mg (for 7 days)  (F) Terfenadine metabolite-Fexofenadine | Clarithrmycin  2x 500 mg | 6 | 29.2 (24-36) | 5/1 |  | 75 (62-89) | 175.5 (170-183) | F: 109.3 | F: 21.2 | F: 153.6 | **Bazett**  21 ms combination  T: 1 ms  C: -1.5 ms |
|  |  | (T) Terfenadine  2x 60 mg (for 7 days)  (F) Terfenadine metabolite-Fexofenadine | (A) Azithromycin  500 mg loading dose, then 1x 250 mg | 6 | 29.2 (19-40) | 4/2 |  | 68.7 (55-80) | 170.5 (157-178) | F: 4.2 | F: 27.8 | F: 2.2 | **Bazett**  1 ms combination  T: 1 ms  A: -16.4 ms |
| Honig 1993^59^ | prospective cohort study,  multiple dose | (T) Terfenadine  120 mg  (single dose)  (F) Terfenadine metabolite-Fexofenadine | (I) Itraconazole  1x 200 mg  (for 7 days) | 6 | 30.5 (24-35) | 2/4 |  | 68 (52-79) | 170.2 (165-183) | F: -70 | F: 147 | F: 30 | **Bazett (mean maximal)**  41 ms combination  T: 14 ms |
| Honig 1993^60^ | prospective cohort study,  multiple dose | (T) Terfenadine  2x 60 mg (for 7 days)  (F) Terfenadine metabolite-Fexofenadine | (Fl) Flucnazole  1x 200 mg for 7 days | 6 | 27.8 (24-35) | 5/1 |  | 75.2 (65-89) | 171 (160-180) | F: 26 | F: 74 | F: 34 | **Bazett**  7 ms combination  T: 0 ms |
| Harris 1995^61^ | double-blind,  multiple dose,  placebo-controlled | (T) Terfenadine  2x 60 mg (for 11 days + 1x 60mg on day 12)  (F) Terfenadine metabolite-Fexofenadine | (A) Azithromycin  1x 500 mg  (on day 8) and 1x 250 mg (from 9-12 day) | 12 in study group/ 12 in control group | Study group: 31.8 (21-42)  Control group: 32.9 (22-44) | 24/0 | Study group:  B 0  H 7  W 5  Control group:  B 2  H 7  W 3 | Study group:  67.1 (49.9-83.5)  Control group: 72.5 (66.7-80.70 |  | F: 64.5 | F: 4.5 | F: 55 | **Bazett**  11 ms combination  T: 10 ms |
| Abernethy 2001^64^ | randomized, double-blind, double-dummy, parallel group, multiple-dose  3 study groups:  (T+Pl, T+N, Pl+N) | (T) Terfenadine  2x 60mg (for 7 days)  (F) Terfenadine metabolite-Fexofenadine | (N) Nefazodon  2x 200 mg  (for 3 days)  2x 300 mg (from 11-15 morning day) | 14 T+N | 33 (22-44) | 9/5 | B 5  W 8  O 1 | 75.8 (53-98.5) | 176 (calculated from BMI) |  |  |  | **Bazett** (R-R >500 ms) or **Fridericia** (R-R <500 ms)  42.4 ms combination  T: 8 ms  N: 10.8 ms |
|  |  |  |  | 13 T+Pl | 33 (21-55) | 6/7 | B 5  W 7  O 1 | 69.0 (51-92.5) | 169 (calculated from BMI) | T: 221  F: 18 |  | T: 430  F: 59 |  |
| Abernethy 2001^64^ | randomized, double-blind, double-dummy, parallel group, multiple-dose  (L+N, L+Pl, Pl+N) | (L) Loratadine  1x 20 mg (for 7 days)  (DL) Loratadine metabolite - Desloratadine | (N) Nefazodon  2x 200 mg (from day 8) then 2x 300 mg (days 11-15) | Pl+N 13 | 33 (21-44) | 7/6 | B 5  W 7  O 1 | 70.5 (49-92) | 169 (calculated from BMI) |  |  |  |  |
|  |  |  |  | L+N 14 | 28 (21-52) | 8/6 | B 2  W 9  O 3 | 71.6 (49-92.5) | 170 (calculated from BMI) | L: 87  DL: 25 |  | L: 131  DL: 43 | **Bazett** (R-R >500 ms) or **Fridericia** (R-R <500 ms)  21.6 ms combination  L: 9.1 ms  N: 10.8 ms |
|  |  |  |  | L+Pl 13 | 32 (21-51) | 10/3 | B 7  W 6 | 71.5 (51-87) | 171 (calculated from BMI) |  |  |  |  |
| Martin 1997^62^ | randomized open-label, two-period, steady-state crossover | (T) Terfenadine  2x 60 mg (for 7 days from day 8)  (F) Terfenadine metabolite-Fexofenadine | (P) Paroxetine 1x 20 mg (for 15 days) | 11 | (21-39) | 11/0 |  | (55-94) |  | T: 1.1  F: -20.6 |  | T: -2.6  F:-18 | **Bazett**  5 ms combination  T: 6 ms |
| Morganroth 1999^66^ | double-blind, randomized, placebo-controlled  4 study groups:  (P, T, S, T+S) | (T) Terfenadine  2x 60 mg  (on days 1-4 for 7 doses) | (S) Sparfloxacin  1x 400 mg on day 1  1x 100 mg on day 2-4 | 88 (4x 22) | 18-49 | 88/0 | B 8  H 8  W 71  O 1 | 60-98.6 |  | Not significant | Not significant | Not significant | **Bazett**  18-22 ms combination  T: 2-6 ms  S: 14-18 ms |
| Akhtar 2002^67^ | balanced randomized open label,  four treatment, four period, four sequence cross-over  4 study groups:  (Pl, T, S, T+S) | (T) Terfenadine  60 mg (single dose) | (S) Sparfloxacin  200 mg (single dose) | 8 |  | 8/0 |  |  |  |  |  |  | **NA**  24.7± 4.5 ms combination  T: 7.5±0.6 ms  S: 14 .1±0.8 ms |
| Bachmann 1997^73^ | randomized, placebo-controlled,  two-way cross-over | (A) Astemizole  30 mg  (single dose on day 4)  (DA) Astemizole metabolite - Desmethylastemizole | (D) Dirithromycin  1x500 mg  (for 10 days) | 18 | Male 24 (21-36)  Female 24.1 (21-44) | 9/9 |  | 77.5 (5.3-101.4) |  | A: -5.3  DA: 0 | A: 10  DA: 16.7 | A: 43.7  DA: 52.4 | **NA**  8 ms combination  A: -19 ms |
| Lefebvre 1997^74^ | randomized, placebo-controlled,  double blind,  cross-over | (A) Astemizole  10 mg  (single dose)  (DA) Astemizole metabolite - Desmethylastemizole | (I) Itraconazole  2x 200 mg  (for 14 days) | 12 | (22-28) | 12/0 | W 12 | (65.4-88.4) |  | A: 9.5  DA: 10.8 | A: 30.8  DA: 598.9 | A: 82.2  DA: 97.5 | **Bazett**  No significant difference in QTc intervals after astemizole during placebo and itraconazole phase |
| Brannan 1995^75^ | prospective, double-blind crossover  3 study groups:  (L, E, L+E) | (L) Loratadine  1x 10 mg  (for 10 days)  (DL) Loratadine metabolite - Desloratadine | (E) Erythromycin  3x 500 mg  (for 10 days) | 24 | 29 (21-40) | 24/0 |  | 76.19 (61.2-95) |  | L: 64.7  DL: 61.3  E:-15 | L: -31.8  DL: -14.8  E:-9.5 | L: 49  DL: 49.8  E: 3.1 | **Bazett**  -3 ms combination  L: - 3 ms  E: 2 ms |
| Carr 1998^78^ | randomized,  cross-over | Loratadine  1x 10 mg  (for 10 days)  (DL) Loratadine metabolite - Desloratadine | (C) Clarithromycin  2x 500 mg  (for 10 days) | 24 | 34 (23-40) | 24/0 | B 2  H 12  W 10 | 72.6 (61-87) |  | L: 76  DL: 64.8  C: not significant | L: 15.4  DL: -31  C: not significant | L: 179.5  DL: 50.3  C: not significant | **Bazett**  4 ms combination  L: 3 ms  C: 11 ms |
| Chaikin 2005^76^ | double-blinded, parallel  group, placebo-control | Loratadine  1x 10 mg  (for 5 days)  (DL) Loratadine metabolite - Desloratadine | (K) Ketoconazole  1x 400 mg  (from day 6 to day 13) | 62 | 25 (18-38) | 62/0 |  | 74.86±1.05 | 178±0.7 | L: 248.2  DL: 82 | L: 18.8  DL: -3.6 | L: 345.9  DL: 94.1 | **Bazett**  10.68 ms combination  L: -0.52 ms  K: 7.52 ms |
| Chaikin 2005^76^ | third-party-blind, parallel  group, placebo-control | (E) Ebastine  2x 20 mg  (for 5 days)  (CE) Ebastine metabolite – carebastine | (K) Ketoconazole  1x 400 mg  (from day 6 to day 13) | 55 | 31.7 (21-24)? | 55/0 |  | 77.5±1.24 | 175±0.9 | E: 1472  CE: 11.5 | E: 77.7  CE: 241.7 | E: 4149.8  CE: 44 | **Bazett**  12.21 ms combination  E: -2.2 ms  K: 6.96 ms |
| Kosoglou 2000^77^ | randomized, evaluator-blind, multiple-dose, three-way crossover | (L) Loratadine  1x 10 mg  (for 10 days)  (DL) Loratadine metabolite - Desloratadine | (K) Ketoconazole  2x 200 mg  (for 10 days) | 25 | 32.8 (18-40) | 25/0 |  | 75.1 (61-101.6) |  | L: 171.6  DL; 76.5  K: - 4 | L: 25  DL: 68.9  K: -29 | L: 247  DL: 82.1  K: -4 | **Bazett**  -0.3 ms combination  L: -3.9 ms  K: -1.6 ms |
|  |  | (L) Loratadine  1x 10 mg  (for 10 days)  (DL) Loratadine metabolite - Desloratadine | (C) Cimetidine  4x 300 mg  (for 10 days) | 24 | 33.6 (19-39) | 24/0 |  | 75.7 (62.5-95) |  | L: 72.1  DL: 5.7  C: -6.8 | L: 4.5  DL: 10.3  C: 0 | L: 69.7  DL: 6.3  C: -5.7 | **Bazett**  -2.3 ms combination  L: -5.4 ms  C: -3.2 ms |
| Banfield 2002^79^ | randomized, 2-way crossover, third  party-blind, multiple dose, placebo-controlled | (D) Desloratadine  1x 7.5 mg  (for 10 days) | (K) Ketoconazole  2x 200 mg  (for 10 days) | 24 | 36.9 (19-50) | 12/12 | B 17  W 7 | 76 (58-94) | 172 (calculated from BMI) | DL: 27  3-OH DL: 50 |  | DL: 20.9  3-OH DCL: 89.7 | **NA**  5.4 ms combination  D: 2.3 |
| Glue 2000  [abstract]^82^ | randomized, 2-way crossover, third  party-blind, multiple dose, placebo-controlled | (D) Desloratadine  1x 7.5 mg  (for 10 days) | (E) Erythromycin  3x 500 mg | 24 | (19-46) | 12/12 |  |  |  | DL: 24 |  | DL: 14 | **NA**  Max QTc change 8%, biggest increase 31 ms, max QTc interval 445 ms for combination |
| Gupta 2001^72^ | randomized, placebo-controlled,  parallel-group, third-party-blind,  multiple-dose | (D) Desloratadine  1x 5 mg  (for 7 days) | (A) Azithromycin  1x 500 mg (on day 3) followed by 1x 250  mg for 4 days | 90 | 34.8 (18-46) | 45/45 | B 10  W 80 | 72.6 (48-105) | 24.5 (19-27) | DL: 19.2  A: 40.2  3-OH DL: 13.5 |  | DL: 7.8  A: 19.3  3-OH DL: 2.5 | **Bazett**  -4.2 ms combination  D: -6.3 ms  A: -0.1 ms |
|  |  | (F) Fexofenadine  2x 60  mg  (for 7 days) | (A) Azithromycin  1x 500 mg (on day 3) followed by 1x 250  mg for 4 days |  |  |  |  |  |  | F: 75.4  A: 3.3 |  | F: 71.5  A: -3 | **Bazett**  1.4 ms combination  F: 1.4 ms  A: -0.1 |
| Tyl 2012^83^ | randomized, placebo-controlled,  multiple-dose, triple dummy,  crossover | (B) Bilastine  1x 20 mg  (for 4 days) | (K) Ketoconazole  1x 400 mg  (for 4 days) | 30 | 33 (20-44) | 16/14 | H 29  W 1 | 66.3 (50.3-84.4) | 166 (146-182) | B: 147.6 | B: -3.6 | B: 81.7 | **Individual**  Largest 9.3 ms combination  Not significant; largest 3.5 ms bilastine |
| GASTROINTESTINAL PROKINETIC AGENTS | | | | | | | | | | | | | |
| Van Haarst 1998^92^ | randomized,  2-way crossover,  multiple-dose | (C) Cisapride  4x 10 mg  (for 5 or 10 days) | (Cl) Clarithromycin  2x 500 mg  (for 5 or 10 days) | 12 | 24 (19-31) |  |  |  |  | C: 169  Cl: -2.7 | C: 33.3  Cl: -31.9 | C: 206.1  Cl: 0.3 | **Bazett**  19-23 ms combination  C: 6 ms  Cl: 3 ms |
| Zix 1997^93^ | randomized, triple crossover | (S) Sparfloxacin  1x 400 mg | (C) Cisapride  3x 10 mg  (for 4 days) | 15 | 28±5 | 15/0 |  | 77±8 | 183±4 | S: 10.2 | S: -53.7 | S: 7.4 | **NA**  29 ms combination |
| Zhao 2001^95^ | open-label, three-phase, sequential (concomitant administration for 6 days) | (F) Fluoxetine  1x 20 mg  (for 32 days) | (C) Cisapride  4x 10 mg  (for 7 days) | 12 | 33.8 (18-40) | 12/0 | B 9  W 3 | 77.7 (62.6-95.3) | 177 (160-190.5) | C:-16 |  | C: -16.3 | **Bazett**  1.6 ms combination  C: 5.6 ms  F: 3.5 ms  **Sagie**  2.7 ms combination  C: 0.3 ms  F: 1.2 ms |
| Alderman 2005^96^ | non-randomized, open label | (S) Sertraline  on days 4-29  starting dose 1x 50 mg, titration every third day up to 200 mg (increment 50 mg)  (DS) Sertraline metabolite - N-desmethylsertraline | (C) Cisapride  4x 10 mg on days 1 and 2, 1x 10 mg on day 3,  4x 10 mg on days 25 - 29, and 1x 10 mg on day 30 | 15 | Male 34.4  Female 41.7  (24-45) | 9/6 | H 14  W 1 | Male 78.7 (67-85)  Female 66.6 (60-71) | Male 175.8 (168-185)  Female 160.9 (155-165) | C: -29.1  S: -3.8  DS: 12.2 | C: -35.9  S: -4.9  DS: 5.5 | C: 6.7  S: 18.9  DS: 21.2 | **Hodges**  1 ms combination (maximal positive change; after 0.5 h)  -7 ms (maximal negative change; after 4 h) |
| Katoh 2003^27^ | prospective | (M) Mosapride  3x 5 mg  (for 14 days) | (E) Erythromycin  4x 300 mg  (for 7 days) | 10 | 23.2±3.04 | 10/0 | J 10 |  |  | M: 56 | M: 0 | M: 83.9 | **Bazett**  12 ms combination (after 1 h)  -2 ms combination (after 2 h)  M: -2 ms |
| Robert 2007^100^ | randomized, placebo controlled,  double-blind, double-dummy, multiple-dose, crossover | (C) Cinitapride  3x 1 mg | (K) Ketoconazole  2x 200 mg | 17 | 34.7± 9.9  Male 32.2±11.5  Female 37.5±7.6 | 9/8 |  | 70.7±17.7  Male 80.3±16.7  Female 59.8±11.8 | 172±10  Male 180±7  Female 164±4 | C: 57.3 | C: 14.3 | C: 63.5 | **Bazett**  2.7 ms combination  C: -0.8 ms  K: 1.8 ms  **Fredericia**  4.4 ms combination  C: -0.4 ms  K: 3.2 ms |
| Boyce 2012^26^ | randomized, placebo-controlled, double- blind, double-dummy crossover | (D) Domperidone  4x 10 mg  (for 7 days) | (K) Ketoconazole  2x 200 mg  (for 7 days) | 24 | 26.6±5.8 (18-39) | 14/10 | B 1 W 23 | 73.5 (53.8-98.8) |  | D: 189  K: 1 | D: -44  K: -5 | D: 353  K: 5 | **Bazett**  12.29 ms combination  D: 2.94 ms  K: 7.00 ms  **Fridericia**  15.90 ms combination  D: 4.20 ms  K: 9.24 ms  **Saggie**  15.06 ms combination  D: 3.75 ms  K: 8.68 ms |
| ANTIEMETICS | | | | | | | | | | | | | |
| Chan 2006^104^ | prospective, randomized, placebo, and positive-controlled (4 study groups: Pl, D, O, D+O) | (D) Droperidol  1.25 mg (single dose) | (O) Ondansetron  4 mg (single dose) | D+O: 99  D: 98  O: 100  Pl: 97 | D+O: 31 (18-45)  D: 35 (18-49)  O: 32 (19-50)  Pl: 37 (18-51) | 0/394 |  | D+O: 49 (33-75)  D: 53 (36-77)  O: 52 (35-73)  Pl: 50 (39-65) |  |  |  |  | **Bazett**  15 ms combination  D: 21 ms  O: 14 ms |
| Charbit 2008^105^ | prospective, double-blind, randomized, placebo-controlled, crossover (3 study groups: D, O, D+O, Pl) | (D) Droperidol  1 mg 10 s iv bolus (single dose) | (O) Ondansetron  4 mg 120 s iv infusion (single dose) | 16 | 29±8 (18-45) | 8/8 |  | 70 (calculated from BMI) | 171±9 | D: 57  O: 23 |  | D: 4  O: 13 | **ΔΔQTc**  **Frediericia**  28 ms combination  D 25: ms  O 17: ms  **Bazett**  43 ms combination  D: 42 ms  O: 24 ms |
| Lee 2014^106^ | prospective | (S) Sevoflurane  (8 vol %, 6 L/min ventilation; vital signs controlled concentration) | (O) Ondansetron  30 min before end of the surgery, ondansetron  0.1 mg*kg^-1^ was given intravenously | 41 | 7.9 ± 2.8 (3-13) | 22/19 |  | 31.3±12.4 | 128.7±18.1 |  |  |  | **Bazett**  25 ms combination  S: 19 ms |
| Chu 2008^108^ | randomized, double- blind, placebo, and positive-control (3 study groups: H, H+DXM, Pl) | (H) Haloperidol  2mg (single dose) | (D) Dexamethasone  5 mg (single dose) | H+D:74  H: 72  D: 74  Pl: 75 | H+D: 42.7  H: 42.8  D: 43.2  Pl: 43.8 | 0/372 |  | H+D: 58.1±9.4  H: 57.3±9.7  D: 58.0±10.4  Pl: 57.0±9.0 | H+D: 154.2±6.9  H: 156.3±5.2  D: 156.2±7.4  Pl: 155.7±7.1 |  |  |  | **NA**  (QTc intervals were below 500 ms for combination and drugs administrated alone) |
| El Azim 2009^107^ | prospective, randomized, placebo and positive-control  (5 study group: M, D, M+D, H, Pl) | (M) Midazolam  50 µg/kg (single dose) | (D) Dexamethasone  8 mg iv (single dose) - alone  or  50 µg/kg (single dose) – in combination | M+D: 30  M: 30  D: 30  H: 30  Pl: 30 | M+D: 36±5  M: 36±5  D: 36±6  H: 35±5  Pl: 34±5 | 0/150 |  | M+D: 80.66±9.33  M: 81.2±9.4  D: 80.70±9.88  H: 80.66±8.9  Pl: 79.93±9.39 | M+D: 171.2±11.85  M: 170.8±11.5  D: 172.3±9.07  H: 172.0±10.7  Pl: 173.2±9.7 |  |  |  | **Bazett**  3 min post-dose  -0.40 ms combination  M: -0.56 ms  D: 2.54 ms  H: 10.54 ms  6 min post-dose  -0.80 ms combination  M: 0.73 ms  D: 4.84 ms  H: 8.57 ms  9 min post-dose  -0.77 ms combination  M: 0.13 ms  D: 3.00 ms  H: 3.67 ms |
| PSYCHOTROPIC DRUGS | | | | | | | | | | | | | |
| Sala 2005^113^ | prospective observational | (M) antipsychotic monotherapy  (haloperidol, olanzapine, risperidone, or quetiapine) | (P) antipsychotic + antidepressant or lithium  (citalopram, escitalopram, sertraline, paroxetine, fluvoxamine, mirtazapine, venlafaxine or clomipramine) | M: 19  P: 19 | M: 45.7±15 (22-77)  P: 45.79 ±12.8 (26-74) | 0/19 |  |  |  |  |  |  | **Bazett**  -1 ms monotherapy  24 ms polytherapy |
| Correll 2009^110^ | case-control | (M) antipsychtic monotherapy  (Olanzapine,  Risperidone,  Quetiapine,  Clozapine,  Ziprasidone) | (P) antipsychotic polytherapy  (Quetiapine,  Aripiprazole,  Risperidone,  Ziprasidone,  Olanzapine) | P: 38  M: 73 | P: 40.9±12.4  M: 44.5±16.7 | P: 25/13  M: 44/29 | P:  W 28  O 10  B 10  M:  W 49  O 24 |  |  |  |  |  | **Bazett**  -5 ms (difference between monotherapy and polytherapy) |
| Harrigan 2004^114^; Laughren 2000^115^ | prospective, open-label, randomized, parallel-group, fixed-sequence | (H) Haloperidol  1x 15 mg (for 27 days) | (K) Ketoconazole (400 mg) + Paroxetine (20 mg) (for 6 days)  FDA  Ketoconazole  2x 200 mg  Paroxetine  1x 20 mg | 32 | 35.7 (20-49)  FDA  Male 33.7 (20-47)  Female 43.0 (35-49) | 25/7 | W 86  B 58  O 39 | Male: 77.9  Female: 75.6 |  | H: 68 |  |  | **Population-based correction**  **QT(ms)/RR^0.35^**  13.2 ms combination  H: 6.9 ms  FDA paper  **Bazzet**  8.9 ms combination  H: 4.7 ms |
|  |  | (Z) Ziprasidone  1x 160 mg (for 31 days) | (K) Ketoconazole (400 mg) (for 5 days)  FDA  Ketoconazole  2x 200 mg | 35 | 37.9 (20-58)  FDA  Male: 38.6 (22-58)  Female: 36.1 (20-47) | 25/10 |  | Male: 85.9  Female: 79.8 |  | Z: 31 |  |  | **Population-based correction**  **QT(ms)/RR^0.35^**  16.7 ms combination  Z: 15.9 ms  FDA paper  **Bazzet**  20.4 ms combination  Z: 20.6 ms |
|  |  | (Q) Quetiapine  1x 750 mg (for 27 days) | (K) Ketoconazole (400 mg) (for 5 days)  FDA  Ketoconazole  2x 200 mg | 29 | 38.7 (26-57)  FDA  Male: 38.1 (26-47)  Female: 40.6 (27-57) | 22/7 |  | Male: 83.9  Female: 87.7 |  | Q: 192 |  |  | **Population-based correction**  **QT(ms)/RR^0.35^**  7.9 ms combination  Q: 5.6 ms  FDA  **Bazett**  19.7 ms combination  Q: 14.5 ms |
|  |  | (O) Olanzapine 1x 20 mg (for 24 days) | (F) Fluvoxamine (100mg) (for 7 days)  FDA  Fluvoxamine  1x 100 mg | 28 | 38.3 (22-58)  FDA  Male: 38.2 (22-58)  Female: 38.6 (25-53) | 20/8 |  | Male: 86.0  Female: 86.2 |  | O: 53 |  |  | **Population-based correction**  **QT(ms)/RR^0.35^**  3 ms combination  O: 1.7 ms  FDA  **Bazett**  5.3 ms combination  O: 6.4 ms |
|  |  | (R) Risperidone  1x 6-8 mg (for 25 days) and 1x 16 mg (for 20 days) | (P) Paroxetine (20 mg) (for 7 days)  FDA  Paroxetine  1x 20 mg | 28 | 38.1 (20-55)  FDA  Male: 38.3 (20-55)  Female: 37.3 (29-47) | 22/6 |  | Male: 84.1  Female: 88.0 |  | R: 111 |  |  | **Population-based correction**  **QT(ms)/RR^0.35^**  2.5 ms combination  R: 3.8 ms  FDA  **Bazett**  3.2 ms combination  R: 10 ms |
|  |  | (T) Thioridazine  1x 300 mg (for 30 days) | (P) Paroxetine (20 mg) (for 7 days)  FDA  Paroxetine  1x 20 mg | 31 | 35.8 (21-48)  FDA  Male: 35.5 (21-48)  Female: 37.3 (30-44) | 25/6 |  | Male: 90.  Female: 87.1 |  | T: 4 |  |  | **Population-based correction**  **QT(ms)/RR^0.35^**  29.8 ms combination  T: 30.4 ms  FDA  **Bazett**  28.0 ms combination  T:35.8 ms |
| Alderman 2005^96^ | nonrandomized, open-label | (P) Pimozide  2 mg (single dose on days 1 and 39) | (S) Sertraline  on days 4-29  starting dose 1x 50 mg, titration every third day up to 200 mg (increment 50 mg)  (DS) Sertraline metabolite - N-desmethylsertraline | 15 | 29.5 (19-41) | 8/7 | H 15 | Male: 70.8  Female: 61.4 | Male: 172.9  Female: 161.1 | P: 35  S: -3.8  DS.: 12.2 | P: 6  S: -4.9  DS: 5.5 | P: 37  S: 18.9  DS: 21.2 | **Hodges**  -4 ms combination (maximal negative change; after 0 h)  7 ms combination (maximal positive change; after 8 h) |
| Desta 1999^116^ | randomized, double-blind placebo-controlled crossover  EM- CYP2D6 extensive metabolizers  PM – CYP2D6 poor metabolizers | (P) Pimozide 1x 6 mg (single dose on day 6) | (Cl) Clarithromycin 2 x 500 mg (for 5 days) | 12  PM: 5  EM: 7) | 28.7 (22-47) | 7/5  PM 4/1  EM:3/4 | W 11  Ch 1 | 73 (59-98) |  | P: 39 (average)  P PM: 54  P EM: 32 | P: 9 (average)  P PM: 8  P EM: 21 | P: 112 (average)  P PM: 90  P EM: 92 | **Fridericia**  10.5 ms combination  P: 9 ms |
| Park 2006^117^ | randomized crossover | (H) Haloperidol  5 mg (single dose on day 7) | (I) Itraconazole  2x 200 mg (for 10 days) | 15  (CYP2D6*1: 8 CYP2D6*10: 7) | 24.5 ± 2.2 | 15/0 | K | 74.1 ± 8.5 |  | H CYP2D6*1: 23  CYP2D6*10: 7 | H  CYP2D6*1: 17 CYP2D6*10: -7 | H  CYP2D6*1: 45  CYP2D6*10: 52 | **Fridericia**  CYP2D6*1:  3.4 ms combination  H: 3.7 ms  CYP2D6*10:  3.2 ms combination  H: 4.1 ms |
| **AGENTS USED IN DRUG DEPENDENCE** | | | | | | | | | | | | | |
| Liu 2007^120^ | randomized, double-blind, sponsor-open, placebo-controlled, parallel-group, multiple-dose | (M) Methadone  1x 32-100 mg for at least 30 days (maintenance therapy) | (V) Voriconazole  2x 400 mg on day 3,  2x 200 mg on days 4-7 | 23  M+V:16  M+P:7 | M+V: 44.0±9.2 (19-54)  M+P: 44.3±6.7 (33-55) | 23/0 | M+V:  B 4  W 12  M+P:  W 7 | M+V: 87.2±18.3 (65.3-135.2)  M+P:  89.9±12.5 (74.8-108.9) |  | (R)-methadone: 30.7  (S)-methadone: 65.4  V: not significant (compared to historical data from a reference study) | (R)-methadone: 20.0  (S)-methadone: 25.0  V: not significant (compared to historical data from a reference study) | (R)-methadone: 47.2  (S)-methadone: 103.4  V: not significant (compared to historical data from a reference study) | **Bazett**, **Fridericia**  Not significant between M+V and M+P |
| Schmittner 2009^119^ | prospective, double-blind, dose-escalation | (M) Methadone  80 mg (maintenance therapy) | (L) Lofexidine  1x 0.4 mg for week 1, escalated each week in 0.2 mg increments up to 1x 1.6 mg for week 8 | 14 | 34.9±5.3 | 8/6 | B 8 |  |  |  |  |  | **Bazett**  M+L: 21.9  (compared to M+Pl) |
| Baker 2006^121^ | prospective, open-label, within-subject study | (B/N) Buprenorphine + Naloxone  (average dose, mean±SD) 16.3±1.1/d, sublingually, for at least 2 weeks | (A) antiretroviral (efavirenz 1x 600 mg for 15 days , nelfinavir 2x 1250 mg for 5 days, delavirdine 2x 600 mg for 7 days, ritonavir 2x 100 mg for 10 days, lopinavir/ritonavir 2x 400/100 mg for 7 days) | 50 | 35.8±7.9 (22-50) | 26/24 | U | 81.4±15.7 (61.5-112.7) |  |  |  |  | **Bazett**  8.15 ms combination  B/N: 1.41 ms  B+delavirdine: 13.12 ms  B+efavirenz: 5.41 ms  B+lopinavir/ritonavir: 6.14 ms  B+nelfinavir: 6.72 ms  B+ritonavir: 9.38 ms |
| Baker 2005 (poster)^122^ | prospective | (B/N) Buprenorphine + Naloxone, a stable dose for at least two weeks | (A) antiretroviral  (efavirenz for 15 days/ nelfinavir for 5 days/ delavirdine for 7 days) | 30  (10 subjects/study) | 35.5±7.8 | 15/15 | B 23  W 7 | 79.8±15.1 |  |  |  |  | **Bazett**  B/N: not significant  B+efavirenz: 0.3 ms  B+nelfinavir: 4.4 ms  B+delavirdine: 9 ms |
| Dackis 2003^123^ | double-blind,  placebo-controlled | (C) Cocaine  30 mg iv | (M) Modafinil  1x 200 mg or 1x 400 mg | 10 | 44 (38-50) | 10/0 | B 9  W 1 | 75 (62-103) |  |  |  |  | NA  Not statisticallysignificant (compared to cocaine+Pl) |
| **ANTIMALARIALS** | | | | | | | | | | | | | |
| Pukrittayakamee 2014^136^ | randomized, open-label, two-arm, crossover, three-period | (P) Primaquine  1x 30 mg | (Cl) Chloroquine  1x 600 mg | 16 | 35.3±8.0 | 4/12 | A 16 | 60.4±6.7 |  | P: 70  Cl: not significant (-2%) | P: -34%  Cl: -34% | P: 123.6  Cl: not significant (0%) | **Fridericia**  6.14 ms combination  P: 1.20 ms  CQ: 6.10 ms |
| Miller 2013^132^ | randomized, double-blind, parallel-group,  3 study groups:  (T+Pl, C+Pl, T+C) | (T) Tafenoquine  1x 450 mg (days 2 – 3, after breakfast) | (Cl) Chloroquine  1x 600 mg (for days 1 -2)  1x 300 mg (for day 3),  after breakfast | T: 20  Cl: 20  T+Cl: 18 | T: 27.0±11.7  Cl: 30.0±12.7  T+Cl: 27.1±7.6 | T: 12/8  Cl: 8/12  T+Cl: 8/10 | T:  B 8  W 11  O 1  C: B 9  W 11  T+Cl:  B 10  W 8 | ≥ 60 |  | Not significant  (T in combination – transient increase: 38% on day 2) | Not significant | Not significant  (Tin combination – transient increase: 24% on day 2) | **Bazett**,  **Fridericia** – maximal changes on day 2 at hour 12:  30.7 ms combination  T: not significant  Cl: 26.7 ms |
| Omoruyi 2007^135^ | randomized, latin-square, crossover, single-dose | (H) Halofantrine  1x 500 mg | (A) Amodiaquine  1x 600 mg  (24 h before H) | 10 | 22-35 | 10/0 | B 10 | 53-72 |  | Not significant | Not significant | Not significant | **NA**  Combination > HF alone |
| Nosten 1993^134^ | prospective study | (M) Mefloquine  1x 25 mg/kg | (H) Halofantrine  3x 8 mg/kg (for 3 days) with food | 114  H: 51  M: 53  M(first)+H: 10 | ≥5 | 72/42 | A |  |  |  |  |  | **NA**  M: not significant  H: significant  MQ+HF: significant  MQ+HF > HF |
| Coyne 1996^127^ | multiple-dose | (M) Mefloquine  250 mg once /week for 5 doses | (H) Halofantrine  4x 500 mg x3 regimen  (7 days after last MQ dose) | 8 | M: 31±8  F: 28±7 | 4/4 | U |  |  |  |  |  | **NA**  99 ms combination  M: 22 ms |
| Gupta 2005^128^ | randomized, parallel group trial, part of a large open-label, 3-arm, randomized ^59^ | (A/P) Atovaquone + proguanil  15 mg/kg/day / 8 mg/kg/day  (for 3 days) | (A) Artesunate  4 mg/kg/day  (for 3 days) | 42 | 24.4 (6-61) | 26/16 | U | 38.7 (14-60) |  |  |  |  | **Bazett**  -6 ms combination  A/P: 11 ms AP |
| Krudstood 2011^130^ | randomized, 2-arm, parallel group | (A) Artesunate  1x100 mg for 3 days;  4 mg/kg (day 0 – day 2) | (M) Mefloquine  1x 200 mg for 3 days;  15 mg/kg (day 1), 10 mg/kg (day 2) | 25  25 | 26.6 (17-50)  28.9 (16-45) | 19/6  22/3 | Thailand | 50.1 (6.26)  51.0 (6.36) |  |  |  |  | ΔQT_c_ on Day 3, all subjects:  **Fridericia**  18 ms (-29 to 98) combination  **Bazett**  5 ms (-49 to 96) combination  New malaria formula  QT/(RR)^0.4^  13 ms (-36 to 97) combination |
| Bindschedler 2000^126^ | randomized, double-blind, double-dummy, parallel group | (CoA) Co-artemether  (atremether + lumefantrine)  6x 80 mg/480 mg /60h | (M) Mefloquine 1000mg (500 + 250 + 250 mg/ 12h) | 45 | 33.7 (19-50) | 45/0 | W 45 | 73.6 (57-86) |  |  |  | A: not significant  M: not significant  L: ~-30-40 | **Bazett**  17±17 ms combination  CoA: 13±11 ms  M: 18±17 ms |
| Aina 2010^125^ | parallel-group | (D) Dihydroartemisinin 1x 120 mg on day 0, 1x 60 mg on days 1-2  (D) alone: 1x120mg on day 0, 1x 60 mg on days 1-6) | (M) Mefloquine  1x 250 mg for 3 days | 20  (D: 10  D+M: 10) | D: 23±1.6 (21-25)  D+M: 24±2.8 (22-29) |  | B | D: 64.8±1.0 (51-78)  D/M: 69.0±4.8 (59-68) |  |  |  |  | **NA**  8.8 ms combination  D: 5 ms |
| Valecha 2001^137^ | randomized, open-label, two-arm | 1. (D) Dihydroartemisinin  1x 2.25 mg/kg for 3 days  2. (A) Artesunate  1x 4 mg/kg for 3 days | 1.(P) Piperaquine  1x18 mg/kg for 3 days  2. (M) Mefloquine  1x 15 mg/kg on day 2, 1x 10 mg/kg on day 3 | 1148  D+P: 767  A+M: 381 | D+P: 25.4±13.3  A+M: 25.8±13.7 | 877/271  D+P: 582/185  A+M: 295/86 | A | D+P: 44.3±15.1  A+M: 44.6±15.1 |  |  |  |  | **Fridericia**  Day 2  22.93 ms D+P combination  14.65 ms A+M combination  Day 7  10.47 ms D+P combination  13.39 ms A+M combination |
| Mytton 2007^133^ | randomized | (D) Dihydroartemisinin  7 mg/kg split into four (0, 8, 24, 48h) or three (0,24,48h) doses. | (P) Piperaquine  55 mg/kg split into four (0, 8, 24, 48h) or three (0,24,48h) doses. | 56 | 18 (median)  6-60 (range) | 44/12 | A |  |  |  |  |  | **Bazett**  2ms at 4 h  14 ms at 52 h  Karen  1 ms at 4 h  Karen: 24 ms at 52 h  **Fridericia**  1 ms at 4 h  29 ms at 52 h |
| Karunajeewa 2003^129^ | open-label | (D) Dihydroartemisinin  Children 2-3 years: 4x 15 mg;  Children 4-6 years: 4x 22.5 mg;  Children 7-10 years:4x 40 mg;  Adults (>16years): 4x 80 mg | (P) Piperaquine phosphate  At times: 0, 6, 24, 32h:  Children 2-3 years: 4x 120 mg;  Children 4-6 years: 4x 180 mg;  Children 7-10 years: 4x 320 mg;  Adults (>16years): 4x 640 mg | 62 (30 children and 32 adults) | Children: 7.5±2.5  Adults: 30.0±11.2  Total: 19.1±14.0 | Children: 16/14  Adults: 15/17  Total: 31/31 | A | Children: 17.3±4.5  Adults: 46.4±6.0  Total: 32.3±15.6 |  |  |  |  | **Bazett**  11 ms after 24 h  7 ms after 48 h  Maximal observed QTc prolongation: 53 ms (female child, 24h) |
| Manning 2014^131^ | two-arm, randomized, double-  blind, placebo-controlled | (D) Dihydroartemisinin  180 mg/d for two days once monthly | (P) Piperaquine  1440 mg/d for two days once monthly | 69  D+P: 47  Pl: 22 | D+P: 26.7±7.51  Pl: 27.27±7.6 | 69/0 | A | D+P: 61.4±7.3  Pl: 57.7±5.4 | D+P: 165.1±5.1  Pl: 164.1±5.5 |  |  |  | **Fridericia**  Electronic:  43 ms combination after 28h  Manual:  51 ms combination after 28h  **Bazett**  Electronic:  39 ms combination after 28h  Manual:  47 ms combination after 28h |
| Lefèvre 2002^138^ | randomized, non-blind, two period crossover | (A/L) Artemether +  Lumefantrine 1x 80 mg/480 mg  (DHA) – artemether metabolite - dihydroartemisinin | (K) Ketoconazole  1x 400 mg (D1),1x 200 mg (D2-D5) | 16 | 29.7 (19-49) |  | W 16 | 78.6 (61.4-87.4) |  | A/L: 124  DHA: 40  L: 26 | Not significant | A/L: 151  DHA: 72  L: 66 | **Bazett**  Not significant  (one exception unrelated to the study medication) |
| VARIA | | | | | | | | | | | | | |
| Saarnivaara 1998^139^ | randomized, four-group study | a. Neostigmine  40 µg/kg  b. Neostigmine  40 µg/kg  c. Edrophonium  200 µg/kg  d. Edrophonium  500 µg/kg | a. Glycopyrronium 8 µg/kg  b. Atropine  20 µg/kg  c. Atropine  300 µg/kg  d. Atropine 7µg/kg | 84 | 34±14 - 37±16 (between groups) |  |  |  |  |  |  |  | **Bazett**  Significant in all four groups; no differences between groups |
| De Kam 2010^141^ | randomized, double-blind, six-period crossover, placebo-controlled | (S) Sugammadex  4 mg/kg iv or 32 mg/kg iv | (R or V) Rocuronium  1.2 mg/kg iv  or  Vecuronium  0.1 mg/kg iv | 83 | Male:  35±7  Female: 34±8 | 41/42 | Male:  B 1  W 40  Female:  A 2  B 1 W 39 | Male: 80±11  Female: 64±9 | Male:  180±8  Female: 167±5 | S(+R): 4.1  S(+V): -0.63 |  |  | **Individual correction**  S 32 mg/kg+R: 6.24 ms  S 32 mg/kg+V: 5.96 ms  S 4 mg/kg: 0.79 ms  S 32 mg/kg: 6.00 ms |
| De Kam 2013^140^ | randomized, double-blind, parallel-group, placebo-controlled,  two-factorial | (P) Propofol  2–6 µg/mL iv (induction); 4 µ/mL (maintenance)  or  (Sev) Sevoflurane  (1.5 minimum  alveolar concentration) | (S) Sugammadex  4 mg/kg | 132  P+Sug:31  P+Pl: 33  Sev+S: 34  Sev+Pl: 34 | P+S:  34.5±10.1  Pro+Pl: 33.0±11.3  Sev+S: 34.3±10.2  Sev+Pl: 34.0±11.7 | P+S: 15/16  P+Pl: 15/18  Sev+S: 16/18  Sev+Pl: 15/19 | P+S:  W 30  O 1  P+Pl:  W 32  O 1  Sev+S:  W 33  O 1  Sev+Pl: W 32  O 2 | P+S: 70.9±9.6  P+Pl: 70.9±12.8  Sev+S: 71.2±12.9  Sev+Pl: 69.8±13.3 |  |  |  |  | **Fridericia**  (after 30 min from S administration)  31.8 ms combination (P+S)  10.2 ms combination (Sev+S)  S: -2.6 ms  P: 33.2 ms  Sev: 14.5 ms |
| Kempsford 2014^142^ | randomized, placebo-controlled, positive-controlled, double-dummy, double-blind, four-way crossover | (F) Fluticasone furoate inh  1x 200 µg for 7 days/  1x 800 µg for 7 days | (V) Vilanterol inh.  1x 25 µg for 7 days/  1x 100 µg for 7 days | 85 | 28 (18-65) | 49/36 | W 60  B 9  A 14  U 2 | 169.7±8.5 | 68.95±11.22 |  |  |  | **Fridericia**  <5 ms combination (200/25 µg and 800/100µg)  **Individual correction**  <5 ms combination (200/25µg)  9.6 ms (maximal mean ΔQT_c_I) combination (800/100µg) |
| Darpo 2013^144^ | randomized, five-period, cross-over, double-blind, placebo-controlled, positive-controlled | (L) Lomitapide  1x 75mg  (or 1x 200 mg, lomitapide alone) | (K) Ketoconazole  1x 200 mg | 56 | 38±9.4 | 37/19 | W 35  B 18  U 3 |  |  | L: 400  K: -9 |  |  | **Individual correction** (Fridericia consistent)  4.8-6.3 ms combination  L: minor changes < 3 ms  K (2-5h postdosing): 5.6-7.5 ms |
| Shehab 2008^143^ | randomized, three-phase, controlled, double-blind, crossover | (L) Losartan 50 mg/day  For 6 weeks | (S) Spironolactone 25 mg/day  For 6 weeks | 8 | 63.7 (47-72) | 4/4 |  |  |  |  |  |  | **Bazett**  Mean difference of QTc dispersion (difference between the maximum and minimum QTc):  27 ms combination  L: 18 ms |
| Zeuli 2013^29^ | retrospective, self-controlled | (F) Fluoroquinolones (levofloxacine, ciprofloxacine)  Individual doses | (A) Azole (voriconazole, fluconazole, posaconazole)  Individual doses | 94  Lev+Vor: 50  Lev+Flu: 37  Lev+Pos: 1  Cip+Vor: 3  Cip+Flu: 3 | 56±13.7 | 56/38 | W 80  B 3  A 2  NatAm 1  O 8 |  |  |  |  |  | **Bazett**  AZ+FQ: 6.1  Lev+Vor: 5.2  Lev+Flu: 9.5  Lev-Pos: -24  Cip-Vor: 10.3  Cip-Flu: -17.0 |

M/F – male/female

NA – not available

Pl - placebo

B – black; H - Hispanic; W - white; J – Japanese; Ch – Chinese; A – Asian; NatAm – native American; U – unspecified; O- other
